# Elucidation of the origin of chiral amplification in discrete molecular polyhedra

**DOI:** 10.1038/s41467-017-02605-x

**Published:** 2018-02-05

**Authors:** Yu Wang, Hongxun Fang, Ionut Tranca, Hang Qu, Xinchang Wang, Albert J. Markvoort, Zhongqun Tian, Xiaoyu Cao

**Affiliations:** 1 State Key Laboratory of Physical Chemistry of Solid Surfaces, College of Chemistry and Chemical Engineering, iChEM and Key Laboratory of Chemical Biology of Fujian Province, Xiamen University, Xiamen, 361005 China; 2 Institute for Complex Molecular Systems and Computational Biology Group, Eindhoven University of Technology, PO Box 513, 5600 MB Eindhoven, The Netherlands

## Abstract

Chiral amplification in molecular self-assembly has profound impact on the recognition and separation of chiroptical materials, biomolecules, and pharmaceuticals. An understanding of how to control this phenomenon is nonetheless restricted by the structural complexity in multicomponent self-assembling systems. Here, we create chiral octahedra incorporating a combination of chiral and achiral vertices and show that their discrete nature makes these octahedra an ideal platform for in-depth investigation of chiral transfer. Through the construction of dynamic combinatorial libraries, the unique possibility to separate and characterise each individual assembly type, density functional theory calculations, and a theoretical equilibrium model, we elucidate that a single chiral unit suffices to control all other units in an octahedron and how this local amplification combined with the distribution of distinct assembly types culminates in the observed overall chiral amplification in the system. Our combined experimental and theoretical strategy can be applied generally to quantify discrete multi-component self-assembling systems.

## Abstract (web summary)

The sergeants-and-soldiers effect, in which a few chiral units induce chirality in a large number of achiral molecules, is difficult to quantify at the molecular level. Here, the authors devise an elegant strategy—combining theory and a system of pure organic polyhedra with chiral and achiral vertices—to understand the mechanism of chiral amplification in discrete molecular assemblies.

## Introduction

Since Pasteur^1^ discovered the spontaneous resolution in ammonium sodium tartrate and stated that life was intimately related to the asymmetry of the universe, the phenomenon of chirality and how it transfers has intrigued scientists^2–5^. Because of its profound impact on life science^6,7^, molecular motors^8^, and practical applications like the asymmetric synthesis^9^ and enantioseparation^10^ of pharmaceuticals, of particular importance is the chiral amplification occurring in molecular reactions and self-assembling systems. Though systematic combined experimental and theoretical investigations have been well established for the chiral amplification in molecular reactions in asymmetric catalysis^11–13^ and autocatalysis^14^, the elucidation of the chiral amplification in self-assembling systems^15–29^ by explicit structural information and rational theoretical modelling remains a big challenge ^30^.

Amplification of chirality in self-assembling systems is usually denoted as the “sergeants-and-soldiers” effect^30,31^, referring to the ability of a few chiral units (the “sergeants”) to control a large number of achiral units (the “soldiers”). Since the pioneering work of Green et al.^31^, many studies have reported on the amplification of chirality and the sergeants-and-soldiers effect in “infinite” systems like helical polymers^32–36^, one-dimensional supramolecular polymers^37–44^, and two-dimensional (2D) supramolecular networks^45–48^. Although these studies provided important prototypes to mimic the chirality transfer in biopolymers and substantially progressed the fabrication of functional soft materials^18,49–51^, the product in such infinite systems usually comprises a mixture composed of polymers (or assemblies) with highly diverse numbers of repeating units—in other words, a “company” containing various kinds of “squads” consisting of distinct numbers of sergeants and soldiers. In contrast, to obtain an in-depth understanding of the amplification of chirality, it is crucial to design systems that provide products with explicit compositions on the molecular level.

An elegant approach to incorporate both chiral and achiral units into discrete assemblies has been described by Reinhoudt and colleagues^52,53,]^, forming hydrogen-bonded double rosettes (squads) that each contains precisely six units of sergeants and/or soldiers. The product (company) includes only limited kinds of discrete assemblies (squads), allowing for the development of kinetic models to fit the experimental data and to simulate chiral amplification in dynamic systems. Another advantage of discrete assemblies over polymers is that they can be well characterised by nuclear magnetic resonance (NMR) and single-crystal X-ray diffraction analyses. Taking these advantages, Nitschke and colleagues^54–57^ systematically studied the amplification of chirality and long-range stereochemical communication in discrete metal–organic cages. However, the thorough investigation of a company consisting of different kinds of squads was hindered by the difficulty in separating these non-covalent interaction-based discrete assemblies.

As a result of the experimental difficulty on the explicit characterisation of multi-component self-assembling systems, the corresponding theoretical studies are limited^58^. Despite few theoretical models^42,52,59–63^, theoretical simulation taking account of molecular information as well as the synergy between experimental and theoretical studies is still to be established.

Herein, we report a strategy to investigate the sergeants-and-soldiers effect not only within a mixture product (company), but also within each kind of discrete assembly (squad) individually. Pure organic octahedra incorporating hexapropyl-truxene faces and both chiral and achiral vertices are constructed through dynamic covalent chemistry. The octahedra containing different numbers of chiral vertices can be separated by chiral high-performance liquid chromatography (HPLC), where the isolated octahedra are sufficiently stable for subsequent NMR and spectroscopic investigations. Such analysis of separated assemblies combined with structural analyses and theoretical simulations allows us to reveal the origin of the strong amplification of chirality in discrete assemblies. Moreover, with a theoretical model for discrete assemblies based on a mass-balance approach, we rationalise the product distributions as a function of the fraction of chiral units, thus unveiling the fundamental mechanisms of the sergeants-and-soldiers effect. The model results perfectly fit our experimental observations and reveal the relationship between the observed sergeants-and-soldiers effect and the relative free energies of the various octahedron types quantitatively.

## Results

### Chiral octahedra with facial rotational patterns

As previously reported^64^, chiral organic octahedra with facial rotational patterns can be constructed from four equivalents of truxene building block and six diamines through dynamic covalent chemistry. In this study, we change the truxene building block by replacing the butyl groups with propyl groups to better separate the mixture of octahedra products.

**Fig. 1:**
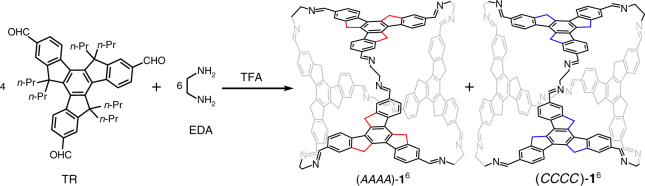
Synthesis of chiral octahedra (*CCCC*)-**1**^6^ and (*AAAA*)-**1**^6^ with facial rotational patterns. Four truxene faces react with six EDA-linked vertices to give tetra-capped octahedral enantiomers. The *C*_3*h*_-symmetric truxene face loses its *σ*_*h*_ mirror symmetry in the octahedra and brings either a clockwise (*C*) or an anticlockwise (*A*) rotation on the exterior faces, as shown with blue or red lines. These configurations of the two octahedra are named after the directionalities of the facial rotations as *CCCC* and *AAAA*. The propyl groups are omitted in the octahedra for clarity

The 5,5,10,10,15,15-hexapropyl-truxene-2,7,12-tricarbaldehyde (TR) was readily synthesised (see Supplementary Fig. 1 and Supplementary Methods) and showed a similar behaviour to that of the previously reported butyl analogues^64^; it can also react with ethylene diamine (EDA) to form the octahedron **1**^6^ with the composition of TR_4_EDA_6_ (Fig. 1), as found by NMR and high-resolution mass spectroscopy (see Supplementary Methods). Single-crystal X-ray diffraction (Supplementary Fig. 2 and Supplementary Tab. 1) and 2D NMR analyses (Supplementary Figs. 3–9) confirmed that the thermodynamic product only contains two enantiomers with homodirectional facial patterns: the (*CCCC*)-**1**^6^ with homodirectional clockwise (*C*) patterns on the exterior faces, and the (*AAAA*)-**1**^6^ with anticlockwise (*A*) patterns. In addition, upon reaction with chiral (*R*,*R*)-diaminocyclohexane (CHDA) instead of EDA, the CHDA vertices dominate the facial directionality of the TR, leading to the diastereoselective synthesis of a merely thermodynamic product (*AAAA*)-**2**^6^ as confirmed by the 2D NMR analyses (Supplementary Figs. 10–16). Density functional theory (DFT) calculations revealed a high similarity between the structures of (*AAAA*)-**2**^6^ and (*AAAA*)-**1**^6^ in terms of the overall conformations and detailed N-C-C-N bonds, angles, and dihedrals on diamine vertices (Supplementary Figs. 17 and 18). DFT calculations also showed that the (*CCCC*)-**1**^5^**2**^1^-(*S*,*S*)-CHDA is indeed the exact mirror image of (*AAAA*)-**1**^5^**2**^1^-(*R*,*R*)-CHDA (Supplementary Fig. 19). However, the energy difference between (*AAAA*)-**1**^5^**2**^1^-(*R*,*R*)-CHDA and (*CCCC*)-**1**^5^**2**^1^-(*R*,*R*)-CHDA is approximately 71 kJ mol^−1^. This large difference is consistent with the absence of the *CCCC*-diastereomers in our experiments.

### Sergeants-and-soldiers effect in a company of mixed squads

**Fig. 2:**
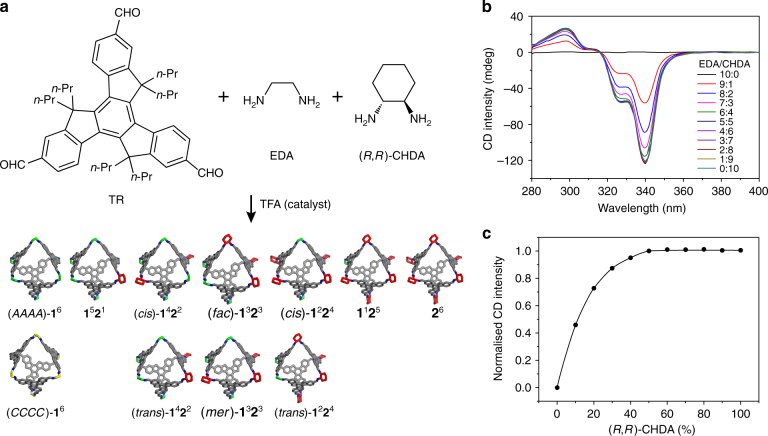
Sergeants-and-soldiers effect in octahedra mixtures. **a** Co-reaction of two different diamine vertices EDA and (*R*,*R*)-CHDA leads to a mixture of molecular octahedra. **b** CD spectra of equilibrium mixtures in toluene at 25 °C for various ratios of EDA to CHDA. **c** Plot of normalised CD intensities at 340 nm vs. the molar fraction of (*R,R*)-CHDA. The red vertices present (*R*,*R*)-CHDA-linked vertices; the yellow and green vertices present EDA-linked vertices in the gauche conformations with dihedral angles of c.a. 60° and c.a. −60°, respectively

We further employed mixtures of achiral EDA and chiral CHDA to form new octahedra with mixed vertices. EDA and CHDA in various ratios (10:0, 9:1,…, 0:10) were mixed with TR and catalytic trifluoroacetic acid (TFA) to form dynamic libraries^65,66^ in toluene (see Supplementary Tab. 2 for detail). The dynamic libraries were immersed in a thermostated bath at 60 °C for 48 h, leading to equilibrium distributions of mixed products incorporating both EDA-linked and CHDA-linked vertices in a single octahedron (Fig. 2a). These octahedra are designated as **1**^*n*^**2**^*m*^, where *n* and *m* represent the number of EDA-linked and CHDA-linked vertices, respectively.

Circular dichroism (CD) spectra of the product mixtures were measured after thermodynamic equilibrium was reached (Fig. 2b). The mixtures with different EDA/CHDA ratios exhibit similar CD spectra with increasing intensities upon increasing fraction of CHDA. The plot of the relative CD intensity (measured at 340 nm) as a function of the molar percentage of chiral CHDA-linked vertices clearly shows a nonlinear chiral amplification upon the increase of the fraction of CHDA (Fig. 2c).

The observed amplification in chiroptical response in truxene octahedra suggests the chiral CHDA can regulate the achiral EDA. This phenomenon is similar to the chiral amplification in some other discrete assemblies formed by hydrogen bonds^52,53^ or metal–organic coordinations ^54–57^.

Regarding the achiral components (EDA) as soldiers and chiral components (CHDA) as sergeants, each discrete assembly, i.e., individual octahedron, can be viewed as a squad and the equilibrium distributions of mixed products can be considered as a company containing various types of squads. All studies on discrete assemblies to date have only revealed the average sergeants-and-soldiers effect in a company, which incorporates various types of squads. To understand the sergeants-and-soldiers effect in discrete assemblies in depth, it is necessary to scrutinise the distinct squads rather than the integrated company.

### Sergeants-and-soldiers effect in isolated octahedra squads

**Fig. 3:**
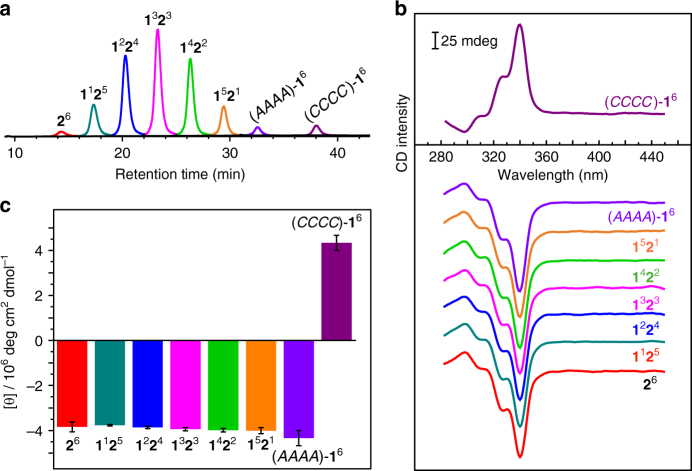
Analysis of octahedra types formed at 1:1 ratio of EDA to CHDA.** a** HPLC spectrum of the equilibrium product. **b** CD spectra of the isolated octahedra types in toluene (offsetted). **c** CD intensities of the isolated octahedra types at 340 nm. Error bars indicate the CD intensity differences obtained from two parallel experiments

Due to the rigidity of the octahedra and the relative stability of imine bonds, we are able to separate the octahedra based on their composition as well as their configuration by chiral HPLC^65^. Eight fractions were found in the mixed equilibrium product containing 50% CHDA (Fig. 3a). These fractions were isolated and individually characterised by mass, CD, and NMR spectroscopies. The matrix-assisted laser desorption ionisation time-of-flight mass spectra of the first six fractions (Supplementary Fig. 20) confirmed their [4 + 6] compositions corresponding to the octahedra **2**^6^, **1**^1^**2**^5^, **1**^2^**2**^4^, **1**^3^**2**^3^, **1**^4^**2**^2^, and **1**^5^**2**^1^, respectively, whereas both of the remaining two fractions matched the composition of octahedron **1**^6^. Although there are possible stereoisomers for octahedra **1**^2^**2**^4^, **1**^3^**2**^3^, and **1**^4^**2**^2^ as shown in Fig. 2a, we did not observe any sign of corresponding peak splitting in the HPLC spectra.

CD spectra of the first six octahedra **1**^*n*^**2**^*m*^ ($0 \le n \le 5,\,m = 6 - n$) and the seventh fraction with the composition of **1**^6^ are almost identical (Fig. 3b, c). According to our previous study^64^ and ZINDO/S simulation (Supplementary Figs. 21 and 22), the CD spectra are strongly dependent on the facial configuration rather than the vertex components, and the octahedra with different facial configurations (i.e., *AAAA*, *AAAC*, *AACC*, *ACCC*, and *CCCC*) exhibit considerably different CD spectra. Therefore, all six octahedra **1**^*n*^**2**^*m*^ ($0 \le n \le 5,\,m = 6 - n$) and the octahedra **1**^6^ in the seventh fraction are in the same facial configuration, i.e., the *AAAA* as in the (*AAAA*)-**2**^6^. And the octahedra in the eighth fraction can be accordingly assigned as (*CCCC*)-**1**^6^, since it exhibits a mirror-like CD spectrum to the (*AAAA*)-**1**^6^ in the seventh fraction.

**Fig. 4:**
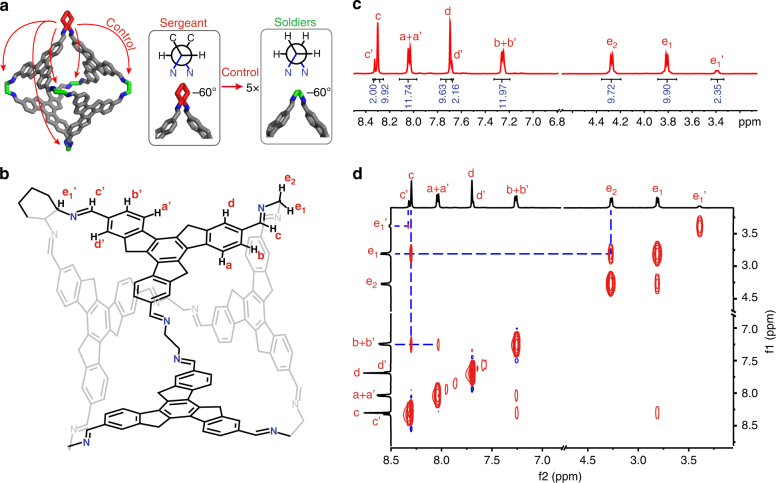
NMR analysis of **1**^5^**2**^1^ to reveal the chirality transfer inside single octahedra. **a** Schematic of an **1**^5^**2**^1^ octahedron shows that one (*R*,*R*)-CHDA sergeant is sufficient to fully control all EDA soldiers in the octahedron squad. **b** Structure of **1**^5^**2**^1^, with propyl groups omitted for clarity and protons labelled. **c** ^1^H NMR and **d** NOE spectrum of **1**^5^**2**^1^, with peaks labelled corresponding to the proton labelling in (**b**)

Every octahedra containing a CHDA-linked vertex has the same *AAAA* configuration, hence all EDA-linked vertices in these octahedra are in the gauche conformation with a dihedral angle of c.a. −60°, as shown in the Fig. 2a. This indicates a strong geometrical control of CHDA sergeants: just a single CHDA-linked vertex (sergeant) suffices to control the remaining EDA-linked vertices (soldiers) in any octahedron (squad), as illustrated in Fig. 4a for a **1**^5^**2**^1^ octahedron.

### Structural basis of chiral amplification in single octahedron

Further understanding of the strong leadership of the CHDA sergeant was revealed by NMR investigation. As a representative example, the ^1^H NMR spectrum of **1**^5^**2**^1^ (Fig. 4b, c) exhibits two adjacent single peaks for the imine protons on a CHDA-linked vertex ($\rm{H}^{{\mathrm{c}}\prime }$, 8.32 ppm) or on an EDA-linked vertex (H^c^, 8.30 ppm). The ratio between the peak areas of $\mathrm{H}^{{\mathrm{c}}\prime }$ and H^c^ is 1:5, in accordance with the number of CHDA- and EDA-linked vertices. Being close to the imine bond on a vertex, the H^d^ on the truxene backbone is also influenced by the CHDA-linked vertex, giving rise to two single peaks in the ratio of 1:5 as well. In contrast, the other two protons on the truxene backbone (H^a^ and H^b^) are less influenced, and they only generate two doublets because of their spin–spin coupling to each other.

Except for the influence of the CHDA-linked vertex, the overall spectrum reveals only a single set of peaks of protons on the truxene backbone, suggesting the truxene faces of **1**^5^**2**^1^ are located in a *T*-symmetry with the facial configuration of *AAAA* or *CCCC*^64,67^. Otherwise, the resonances would further split into three sets (for *C*_3_-symmetric *CCCA* and *CAAA*) or six sets (*C*_2_-symmetric *CCAA*) due to different facial configurations^64^. Considering the CD analysis, the configuration of **1**^5^**2**^1^ is assigned to be *AAAA*. The ^1^H NMR spectra of the octahedra **1**^*n*^**2**^*m*^ ($0 \le n \le 5,\,m = 6 - n$) are rather similar (Supplementary Fig. 23), corroborating that all of the six octahedra with CHDA-linked vertices have the same *AAAA* facial configuration.

The nuclear Overhauser effect (NOE) crosspeak between H^c^ and H^b^ (instead of H^d^) shown in the NOE spectrum of **1**^5^**2**^1^ (Fig. 4b, d) indicates that all imine bonds rotate in the same anticlockwise direction as the sp^3^ carbons of the truxene core. The NOE crosspeak between H^c^ and H^e1^ (instead of H^e2^) indicates that all five EDA-linked vertices are in the same gauche conformation like the CHDA-linked vertex. The structural rigidity of truxene octahedra and the consistency of vertex conformation are also confirmed by the NOE spectra (Fig. 4d) and the single-crystal analysis of **1**^6^ (Supplementary Fig. 2). We presume the structural rigidity and the conformational consistency are crucial to the efficient chiral amplification inside the octahedra.

To shed light on the conformational consistency of the EDA-linked vertices, we calculated the free energies of different conformers of (*AAAA*)-**1**^5^**2**^1^ using DFT calculations. The (*AAAA*)-**1**^5^**2**^1^ conformer with all EDA-linked vertices in c.a. −60° gauche conformation has a much lower energy than any other conformer. For illustration, the difference in energy between the conformer with all EDA-linked vertices in c.a. −60° gauche conformation and the conformer with three EDA-linked vertices in c.a. 60° gauche conformation is approximately 108 kJ mol^−1^ (Supplementary Fig. 24). To our knowledge, the resulting consistency in vertex conformation is a unique property of truxene octahedra, which is not possessed by other similar organic octahedra. For example, in the TFB_4_EDA_6_ octahedra formed from 1,3,5-triformylbenzene (TFB) and EDA^68,69^, the EDA-linked vertices have been proven to be able to dynamically change between c.a. 60° gauche conformer and c.a. −60° gauche conformer^68^, and both *T*-symmetric and *C*_3_-symmetric TFB_4_EDA_6_ exist in the crystal products ^69^.

### Equilibrium distribution analysis and theoretical model

**Fig. 5:**
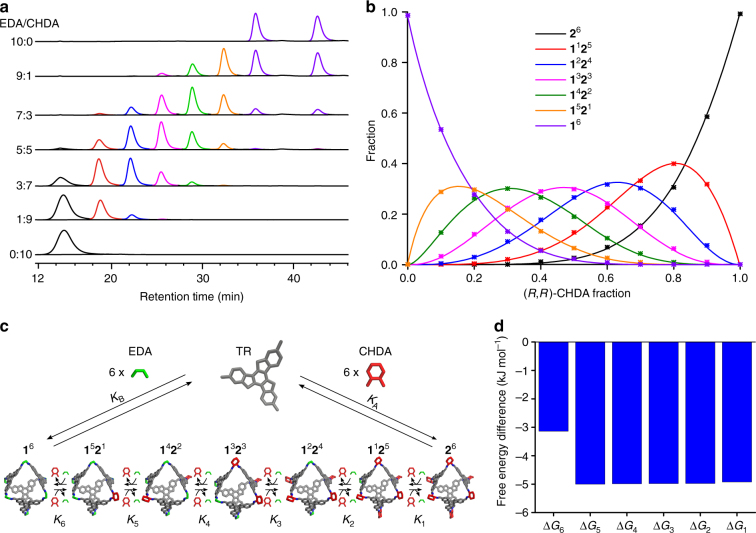
Octahedra equilibrium distributions as a function of the EDA/CHDA ratio. **a** Individual HPLC spectra of the thermodynamic products formed at various ratios of EDA to (*R*,*R*)-CHDA, **b** Fractions of the various octahedra species as derived from the HPLC spectra (symbols) and as fitted with the mass-balance model (lines), **c** Schematic of the mass-balance model, and **d** free energy differences between subsequent octahedra $( K_i = {\mathrm e}^{\Delta {G_i}}/R T)$ corresponding to the fit in (**b**)

To elucidate the distribution of sergeant over the squads, we subsequently analysed the products formed for the different molar fractions of CHDA by HPLC, as shown in Fig. 5a and Supplementary Figs. 25–35. As is evident from Fig. 5b, the **1**^*n*^**2**^*m*^ product distribution is strongly controlled by the molar fraction of CHDA. High CHDA ratios in the mixtures result in predominant formation of the octahedra with high values of *m*, and vice versa. Further investigation of the product distributions with different ratios of chiral units confirmed that the general sergeants-and-soldiers effect in a company is a weighted average of the effects in each squad (Supplementary Fig. 36).

To rationalise the equilibrium distributions of **1**^*n*^**2**^*m*^ product as a function of the molar fraction of CHDA, we devised a mass-balance model as illustrated in Fig. 5c. This model is built on the same principles as earlier thermodynamic models for mixed discrete assemblies^52,60–62^, but differs essentially as it explicitly takes possible differences between the equilibrium constants due to cooperative effects into account. In this model, seven types of octahedra are considered, i.e., a single type for each ratio of EDA-linked and CHDA-linked vertices. **1**^6^ represents both (*AAAA*)-**1**^6^ and (*CCCC*)-**1**^6^, which are assumed to be equally abundant, while the other **1**^*n*^**2**^*m*^ represent all possible conformers with an *AAAA* facial configuration only. The dynamic exchange of CHDA and EDA between the octahedral vertices is described using 6 independent equilibrium constants *K*_*i*_ (1 ≤ *i* ≤ 6), which are related to free energy differences via *K* = e^Δ^^*G*/*RT*^. Whereas possible differences between equilibrium constants were priorly ignored in the absence of data on individual species, our physical separation of the various octahedron types allows to determine them individually. The equilibrium constants allow us to express the equilibrium concentrations of all distinct octahedron types in terms of the equilibrium concentration of **2**^6^:

1 $$\left[ {{\bf 1}^i{\bf 2}^{6 - i}} \right]_{{\rm{eq}}} = \left( {\begin{array}{*{20}{c}} 6 \\ i \end{array}} \right)\left( {\mathop {\prod}\limits_{j = 1}^i K_j} \right)\left( {\frac{{\left[ {{\mathrm{EDA}}} \right]_{{\rm eq}}}}{{\left[ {{\mathrm{CHDA}}} \right]_{{\rm eq}}}}} \right)^i\left[ {{\bf 2}^6} \right]_{{\mathrm{eq}}}.$$

Three mass balances can be derived for the model: (i) the overall TR concentration should equal the equilibrium concentration of free TR plus four times the concentrations of all octahedra types summed, (ii) the overall concentration of EDA should equal the equilibrium concentration of free EDA plus the sum of the concentrations of each octahedra type multiplied by its respective number of EDA-linked vertices, and (iii) the analogous mass balance for CHDA. As detailed in the supporting information (Supplementary Eqs. 12-14), these three mass balances in combination with Eq. 1 allow to calculate the equilibrium concentrations of all octahedra types for a given set of equilibrium constants *K*_*i*_ and overall concentrations of TR, CHDA, and EDA.

Octahedron distributions calculated as a function of the molar fraction CHDA can subsequently be compared to the experimental data (summarised in Supplementary Tab. 3). Best fits of the octahedron distributions and CD intensity are shown in Fig. 5b and Supplementary Fig. 37, respectively. These fits were obtained with the equilibrium constants corresponding to the free energy differences as shown in Fig. 5d and Supplementary Fig. 38. This shows that **2**^6^ has a lower free energy than **1**^6^. In addition, it shows that the free energy gains upon insertion of the second, third, fourth, fifth, and sixth CHDA-linked vertex are all rather similar, whereas the free energy gain upon insertion of the first CHDA-linked vertex is approximately 2 kJ mol^−1^ smaller. That **2**^6^ should have a lower free energy than **1**^6^ is also corroborated by the experimental HPLC results (Supplementary Tab. 3 and Supplementary Fig. 39); these indicate that for the same excess of major diamine vertex, the fraction of **2**^6^ is always higher than that of **1**^6^ and the free CHDA concentration is always lower than the free EDA concentration.

The relative free energies upon the exchange between CHDA and EDA vertices as predicted by the mass-balance model are also in accordance with DFT calculations of the various types of octahedra (Supplementary Fig. 40 and Supplementary Tab. 4). These calculations further showed that upon the exchange between CHDA and EDA vertices only minor conformational changes (Supplementary Figs. 17 and 18 and Supplementary Tab. 5) and changes in electron distributions (Supplementary Fig. 41 and Supplementary Tab. 6) occur. Nevertheless, analysis of the density of states (Supplementary Figs. 42 and 43) showed that free CHDA has some states closer to the Fermi level than free EDA has, indicating that CHDA is slightly more reactive. In addition, both integrated crystal orbital Hamiltonian population and integrated crystal orbital overlap population analyses suggest that the N-C bond is slightly stronger for the CHDA-TR case than for the EDA-TR case (Supplementary Figs. 44 and 45 and Supplementary Tab. 7). Together, these findings explain for the slight preference of CHDA vertices over EDA vertices as observed in the experimental and modelling results.

## Discussion

We have developed a strategy that permits in-depth investigation of the amplification of chirality in discrete molecular assemblies, both from an experimental and a theoretical perspective. Chiral octahedra incorporating a combination of chiral and achiral vertices have been constructed through dynamic covalent chemistry as an experimental model. The product mixtures were first investigated by CD spectroscopy to show a non-linear amplification of CD intensities upon the increase of the fraction chiral vertices; i.e., a notable sergeants-and-soldiers effect in an integrated company. Subsequently, the sergeants-and-soldiers effects within the individual kinds of octahedra (squads) were investigated by separating all octahedron types by chiral HPLC, providing much more explicit information on chirality amplification than by the conventional investigation of mixtures. All octahedra containing one or more chiral vertices exhibit the same CD spectrum as the octahedron containing pure chiral vertices, indicative that one chiral vertex (sergeant) suffices to control the conformation of all achiral vertices (soldiers) in an octahedron (squad). NMR analyses and DFT calculations attribute this strong chiral amplification within octahedra to the structural rigidity of truxene faces and interactions between the propyl arms on truxene. Furthermore, a newly developed mass-balance model for mixed octahedra perfectly fitted the observed sergeants-and-soldiers effects. With this model the equilibrium distribution of the various octahedra, i.e., the distribution of the sergeants over the squads, could be rationalised as a deviation of the statistical distribution due to small free energy differences between the octahedra. DFT calculations attributed these differences in free energy to minor conformational differences between the octahedra and a slightly stronger binding of CHDA over EDA. As such, we presented a combined experimental and theoretical strategy that can be applied more generally to quantify small differences in association energy in discrete multicomponent systems. Through the design of a suitable experimental system and complementary theoretical equilibrium model we thus revealed the origin of chiral amplification in discrete molecular polyhedra, which may provide fundamental insights into the transfer of chirality in supramolecular systems as well.

## Methods

### Synthesis

TR building block was readily synthesised from truxene in three steps with high yields (experimental and characterisation details can be found in the Supplementary Methods). Stock solutions of TR (3.2 mM), EDA (9.6 mM), (*R,R*)-CHDA (9.6 mM), and TFA (19.2 mM) in toluene were mixed at certain volume ratios to give the samples A to K, with concentrations of the various species as detailed in Supplementary Tab. 2. The mixtures were then immersed in a thermostated bath at 60 °C for 48 h to reach equilibrium.

### NMR and MS characterisation

^1^H and ^13^C NMR spectra were recorded on a Bruker AVIII-500 spectrometer (500 MHz) in deuterated dichloromethane and are reported relative to residual solvent signals. Matrix-assisted laser desorption ionisation time-of-flight mass spectra were collected on a Bruker microflex LT-MS with 2,4,6-trihydrotyacetophenane (0.05 M in methanol) as matrix. High-resolution mass spectra were collected on a Bruker En Apex Ultra 7.0T FT-MS.

### Single-crystal X-ray diffraction

Single-crystal X-ray diffraction data were collected on a Rigaku SuperNova X-Ray single crystal diffractometer using Cu K*α* (*λ* = 1.54184 Å) micro-focus X-ray sources at 100 K. The raw data were collected and reduced using the CrysAlisPro software package, while the structures were solved by direct methods using the SHELXS program and refined with the SHELXL program. Solution and refinement procedures are presented in the Supplementary Methods and specific details are compiled in Supplementary Tab. 1.

### HPLC and CD characterisation

HPLC analyses were performed on a Shimadzu LC-16A instrument at 298 K using a Daicel Chiralcel IE column. A linear gradient elution was employed within 40 min from 5% ethyl acetate to 30% ethyl acetate in *n*-hexane with 4% ethanol and 0.1% diethylamine of total volume at a flow rate of 1 mL  min^−1^. The sample concentration was 400 μM in toluene, and the injection volume was 3 μL. Absorbance of octahedra was monitored at 325 nm. HPLC spectra of the equilibrium products containing different ratios of CHDA are presented in the Supplementary Figs. 25–35. CD spectra were measured in toluene solutions with a JASCO J-810 circular dichroism spectrometer.

### Computational methods

All structures were first optimised by the molecular mechanics method (using COMPASS II force field) and further optimised by the DFT method (using Vienna ab initio Simulation Package (VASP)). The electronic structure and bonding analyses were performed based on the partial density of states, crystal orbital Hamiltonian population, crystal orbital overlap population functions and Bader topological analysis. The CD spectra were calculated at ZINDO semi-empirical level with Gaussian 09. Details on the methods are provided in the Supplementary Methods.

### Model for equilibrium distributions

The model was built based on the mass-balance approach and implemented in Matlab of which we used the lsqnonlin function to solve the non-linear equations. The experimental data used for fitting the model were obtained from HPLC experiments and summarised in Supplementary Tab. 3. Modelling details can be found in the Supplementary Methods.

### Data availability

Crystallographic data in this study were deposited at the Cambridge Crystallographic Data Centre with the accession code (CCDC 1517934). The authors declare that all other data supporting the findings of this study are available from the article and its Supplementary Information files or available from the authors upon reasonable request.

## Electronic supplementary material

Supplementary Information
