# Supplementary material for: Elucidation of the origin of chiral amplification in discrete molecular polyhedra
Source: Nat Commun. 2018 Feb 5;9:488. doi: 10.1038/s41467-017-02605-x (PMC5799371; doi:10.1038/s41467-017-02605-x)
Supplement: Supplementary file 1 — Supplementary Information [file 41467_2017_2605_MOESM1_ESM.pdf]

Supplementary Information for

Elucidation of the origin of chiral amplification in discrete molecular polyhedra

Yu Wang[1†], Hongxun Fang[1†], Ionut Tranca[2], Hang Qu[1], Xinchang Wang[1], Albert J. Markvoort[2*], Zhongqun Tian[1], and Xiaoyu Cao[1*]

[1] *State Key Laboratory of Physical Chemistry of Solid Surfaces, iChEM and College of Chemistry and Chemical Engineering, Xiamen University, Xiamen 361005, China.*

[2] *Institute for Complex Molecular Systems and Computational Biology group, Eindhoven University of Technology, PO Box 513, 5600 MB, Eindhoven, The Netherlands.*

[†] *These authors contributed equally to this work.*

[*] *e-mail: xcao@xmu.edu.cn; a.j.markvoort@tue.nl*

Supplementary Figures

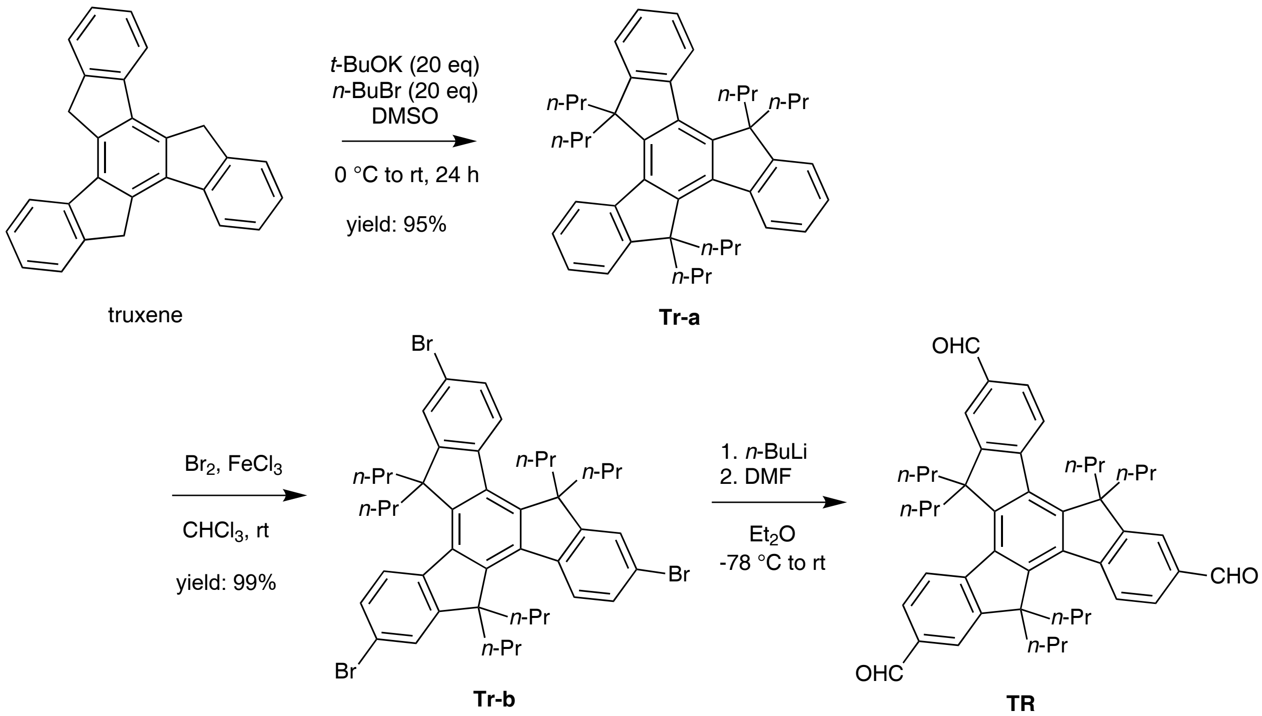

Supplementary Fig. 1. Representative procedures for the synthesis of building block TR.

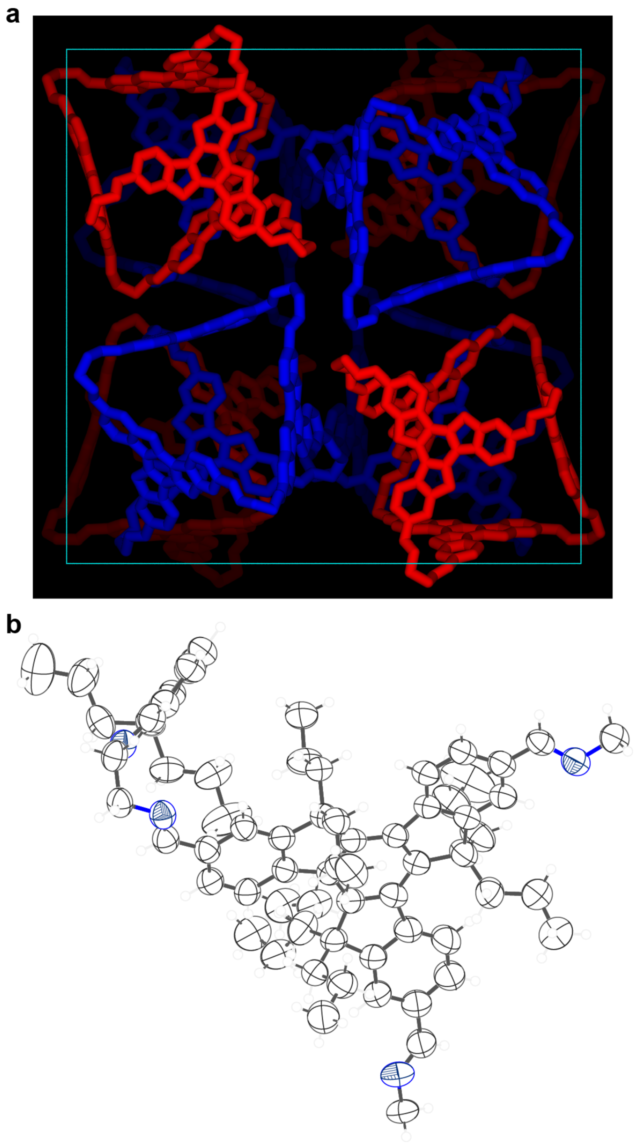

Supplementary Fig. 2. Single crystal structure (a) and the ORTEP drawing (b) of $\mathbf{1}^{6}$. (Displacement ellipsoids for all non-H atoms at the 50% probability level).

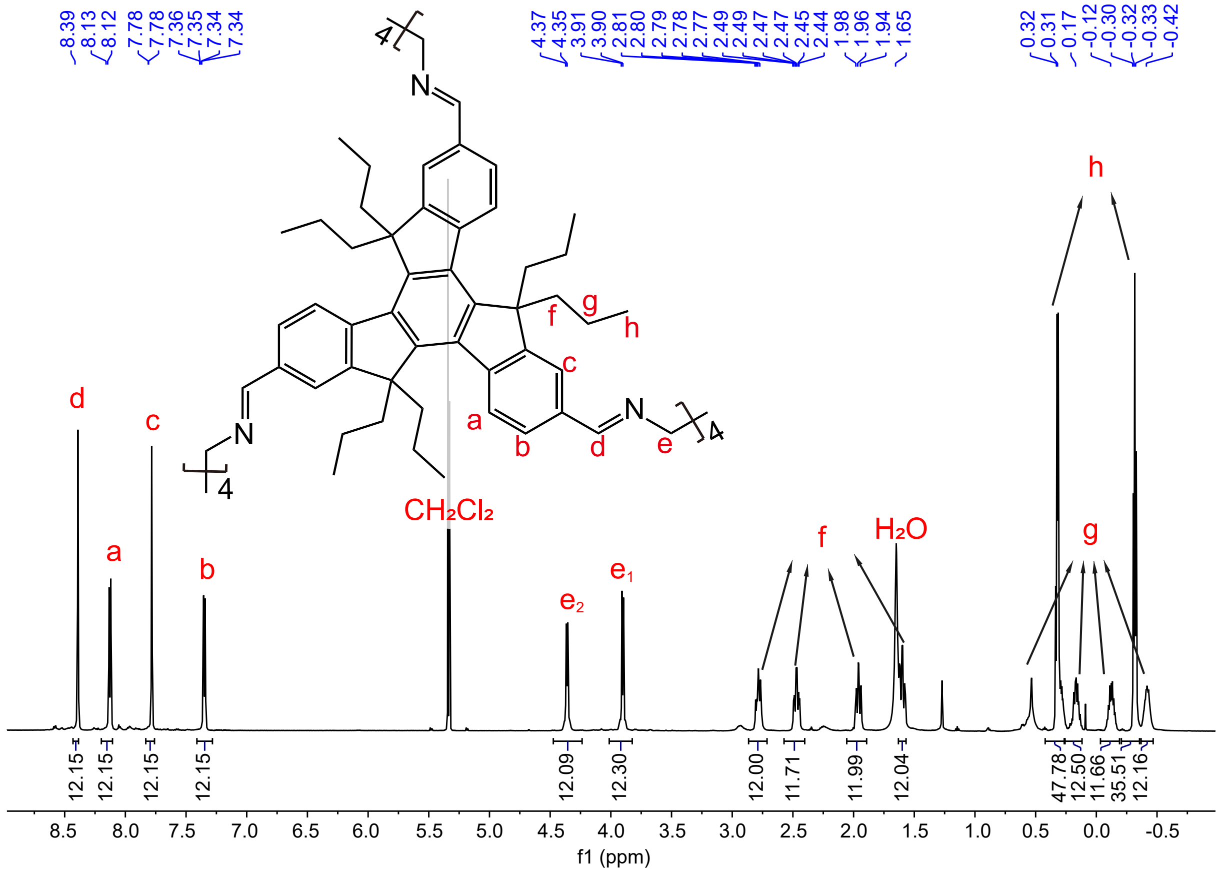

Supplementary Fig. 3. **^{1}H NMR spectrum of 1^6.**

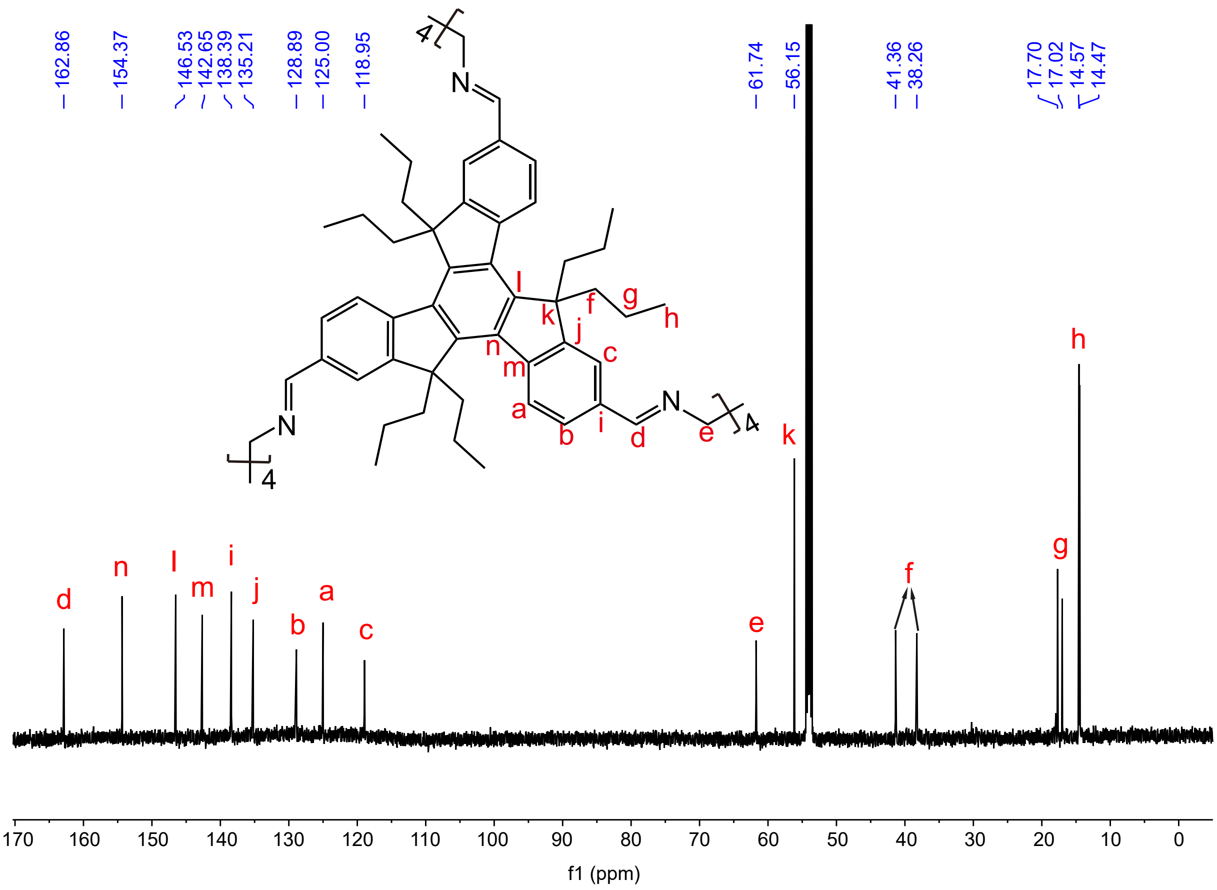

Supplementary Fig. 4. ^{13}**C NMR spectrum of 1**^{6}**.**

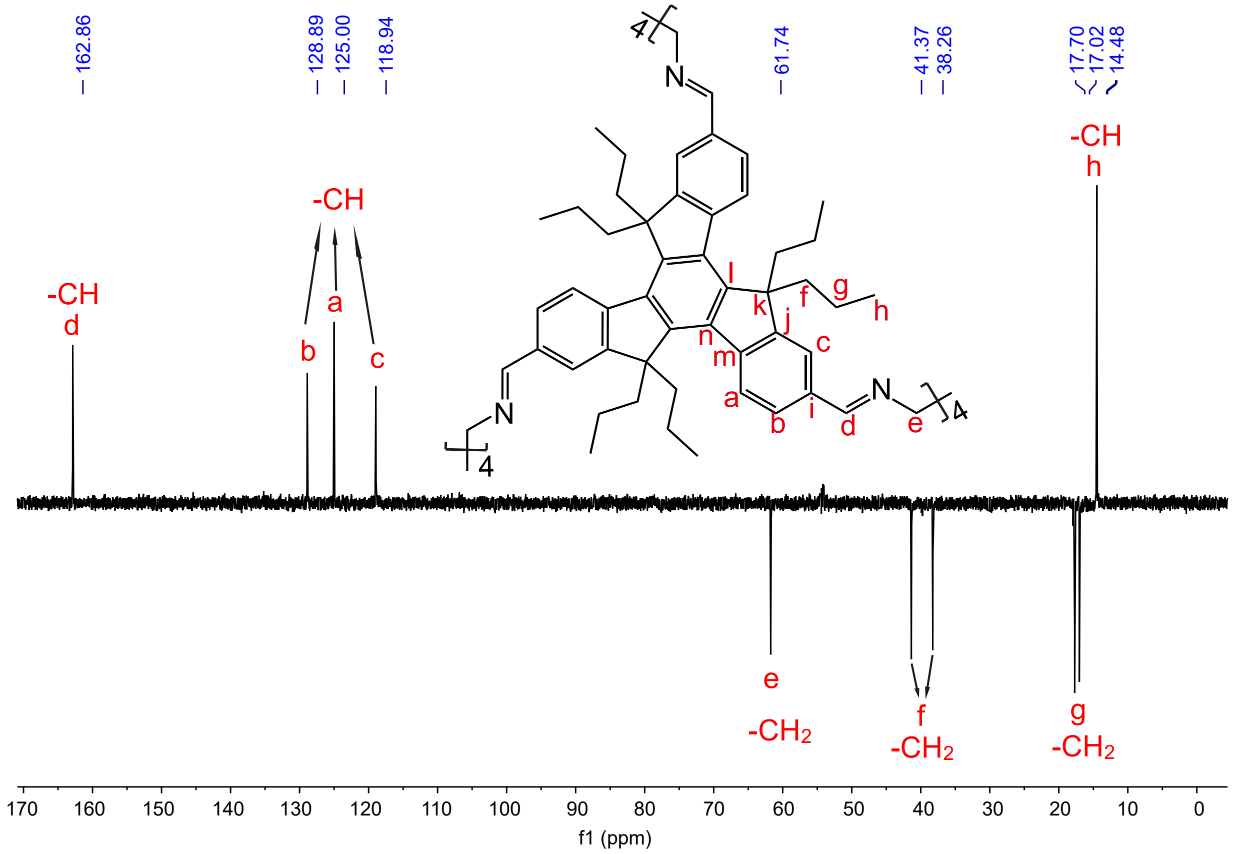

Supplementary Fig. 5. Distortionless Enhancement by Polarization Transfer (DEPT)-135 spectrum of 1^6.

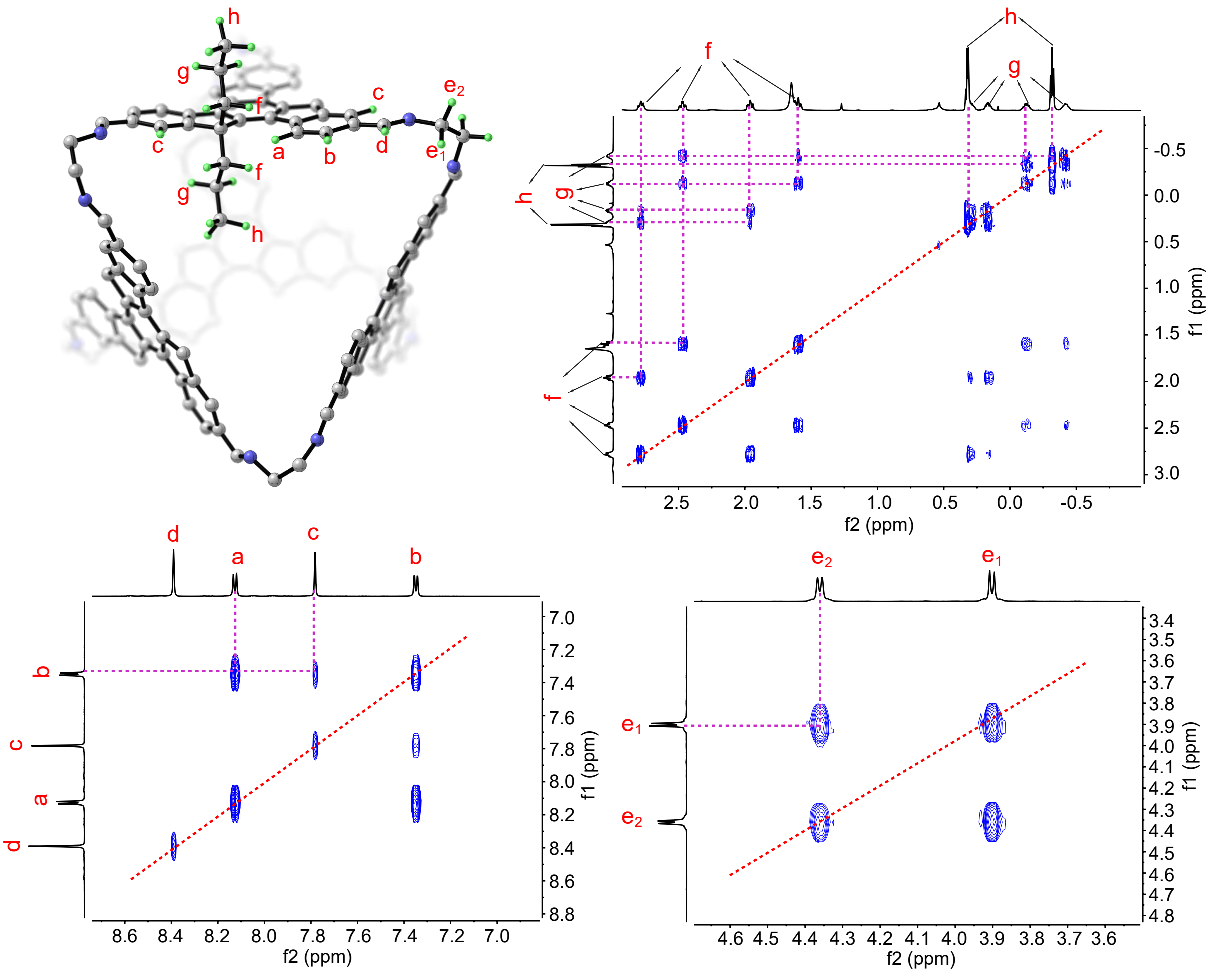

Supplementary Fig. 6. HH correlation spectroscopy (COSY) spectrum of 1^6 (only one propyl chain is shown for clarity).

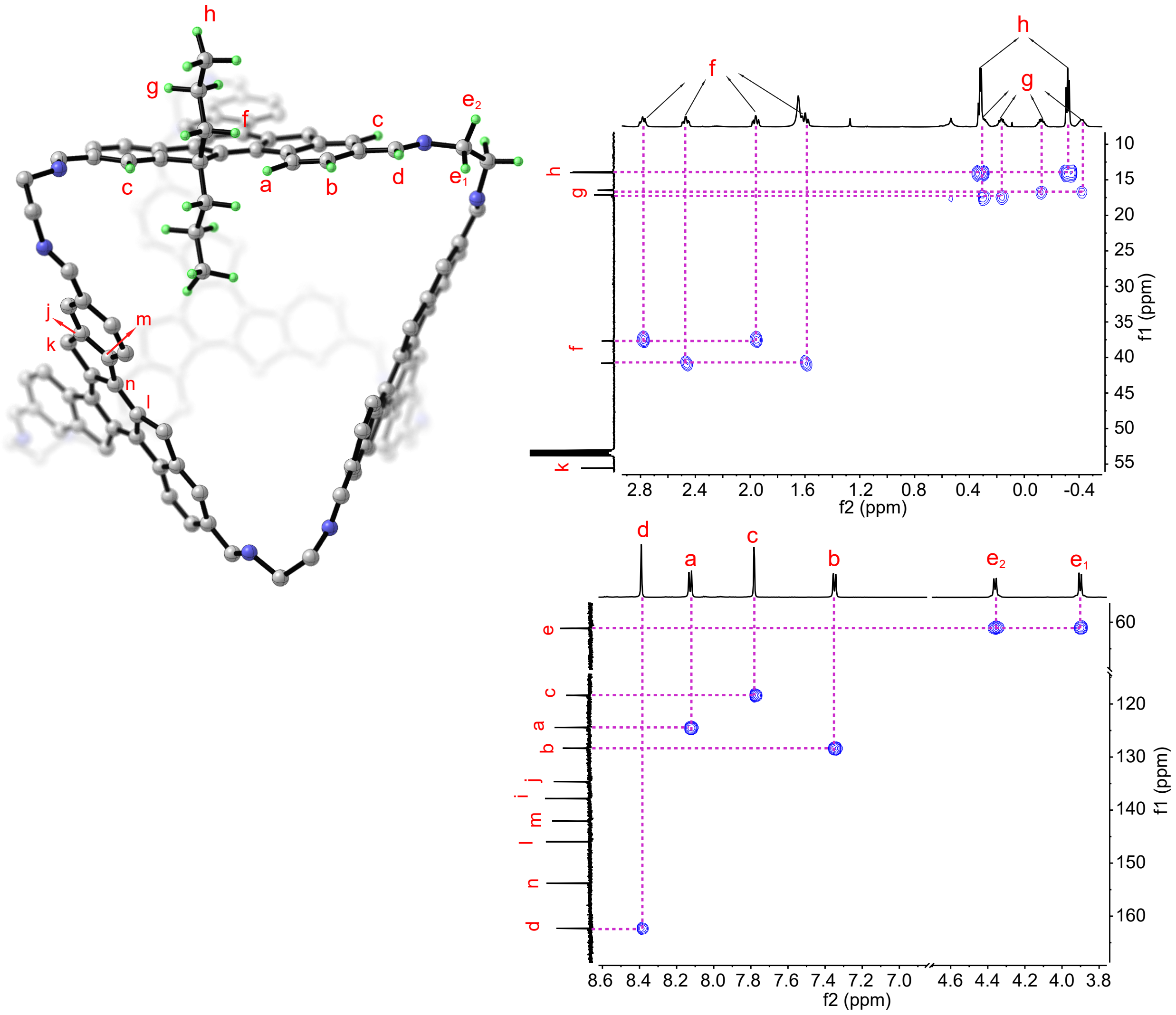

Supplementary Fig. 7. Heteronuclear single-quantum correlation (HSQC) spectrum of 1^6.

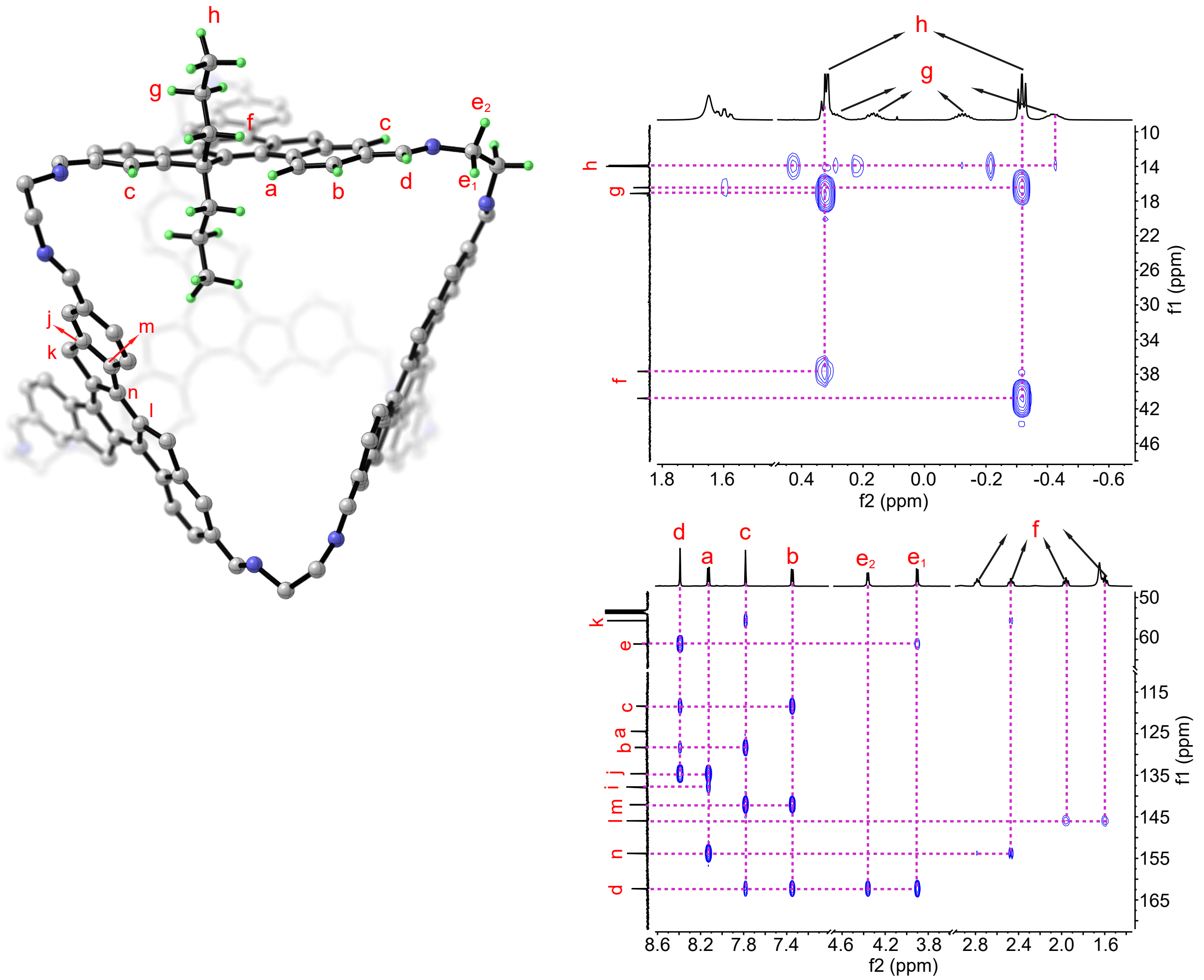

Supplementary Fig. 8. ^{1}**H detected heteronuclear multiple bond correlation (HMBC) spectrum of 1**^{6}**.**

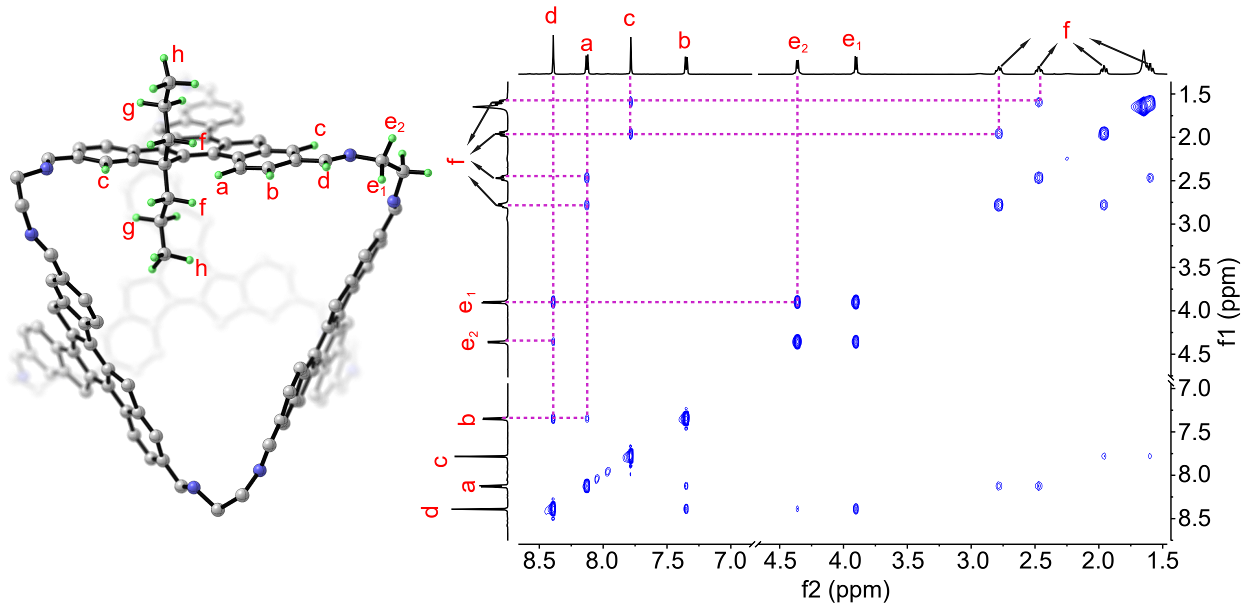

Supplementary Fig. 9. Nuclear Overhauser enhancement spectroscopy (NOESY) spectrum of 1^6.

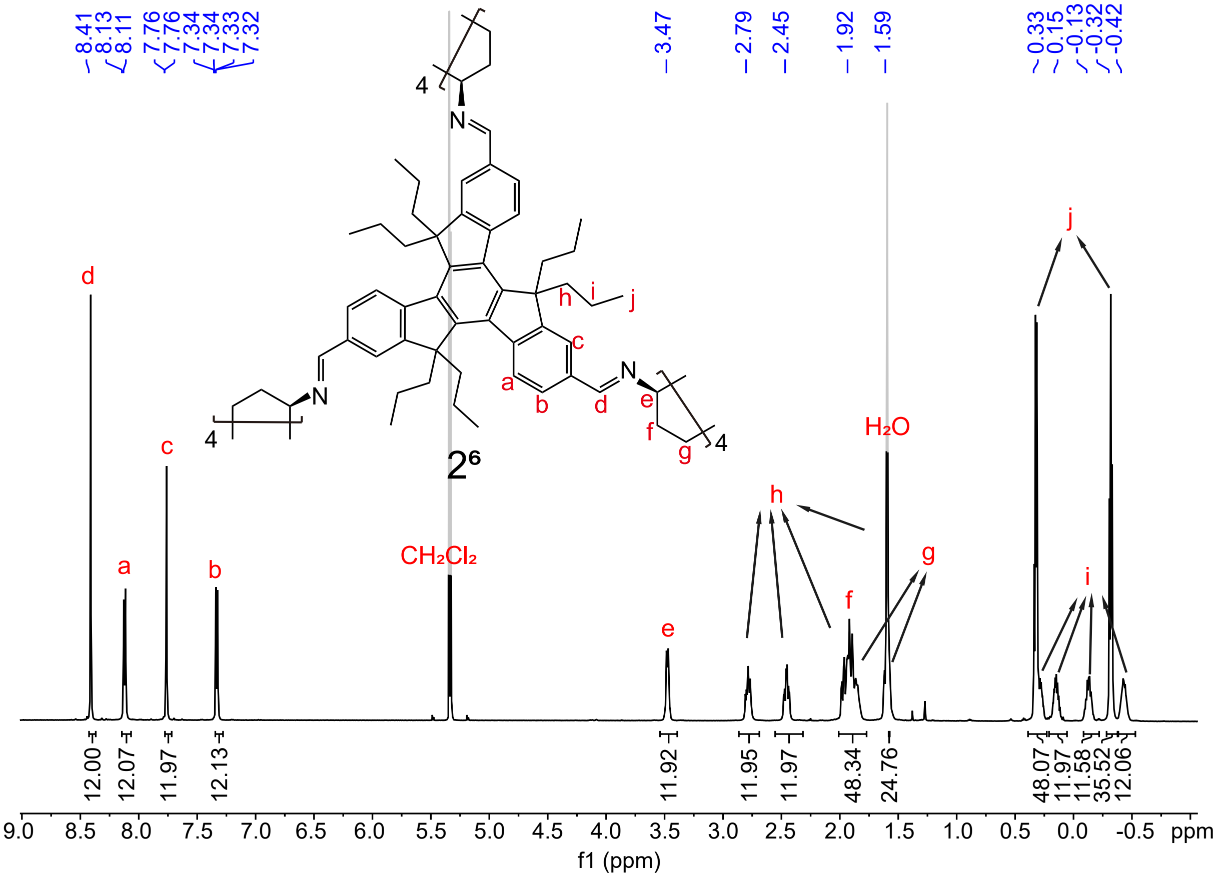

Supplementary Fig. 10. ^{1}**H NMR spectrum of 2**^{6}**.**

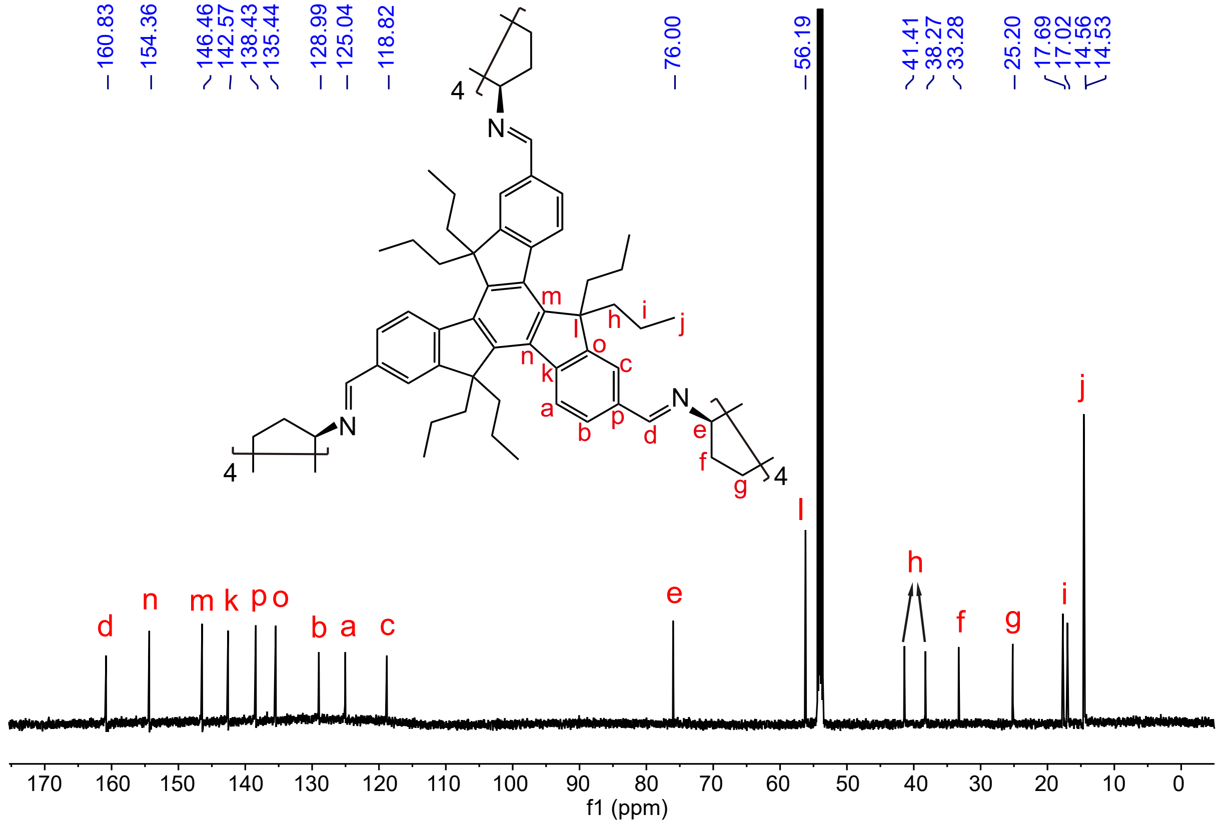

Supplementary Fig. 11. ^{13}**C NMR spectrum of 2**^{6}**.**

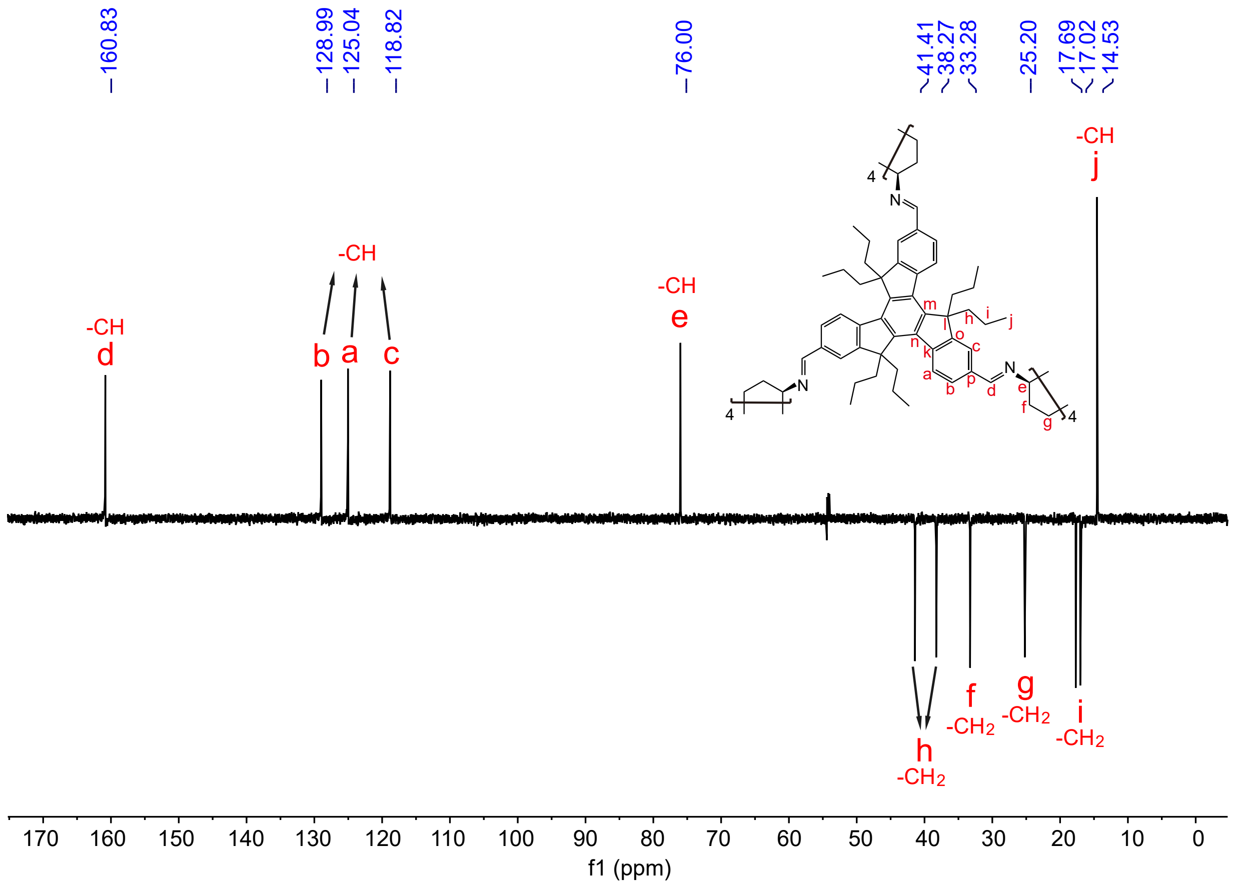

Supplementary Fig. 12. Distortionless Enhancement by Polarization Transfer (DEPT)-135 spectrum of 2[6].

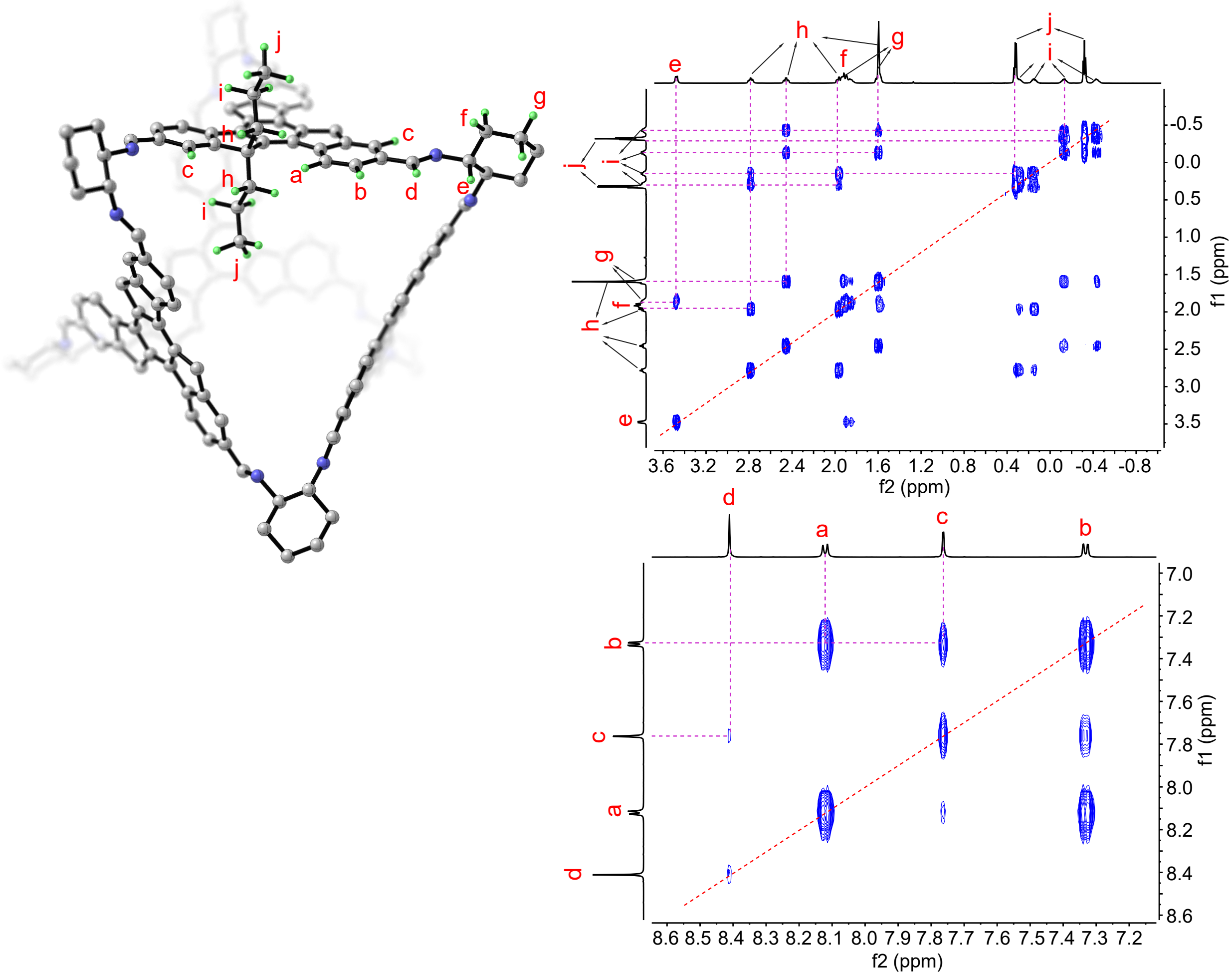

Supplementary Fig. 13. HH correlation spectroscopy (COSY) spectrum of 2^6.

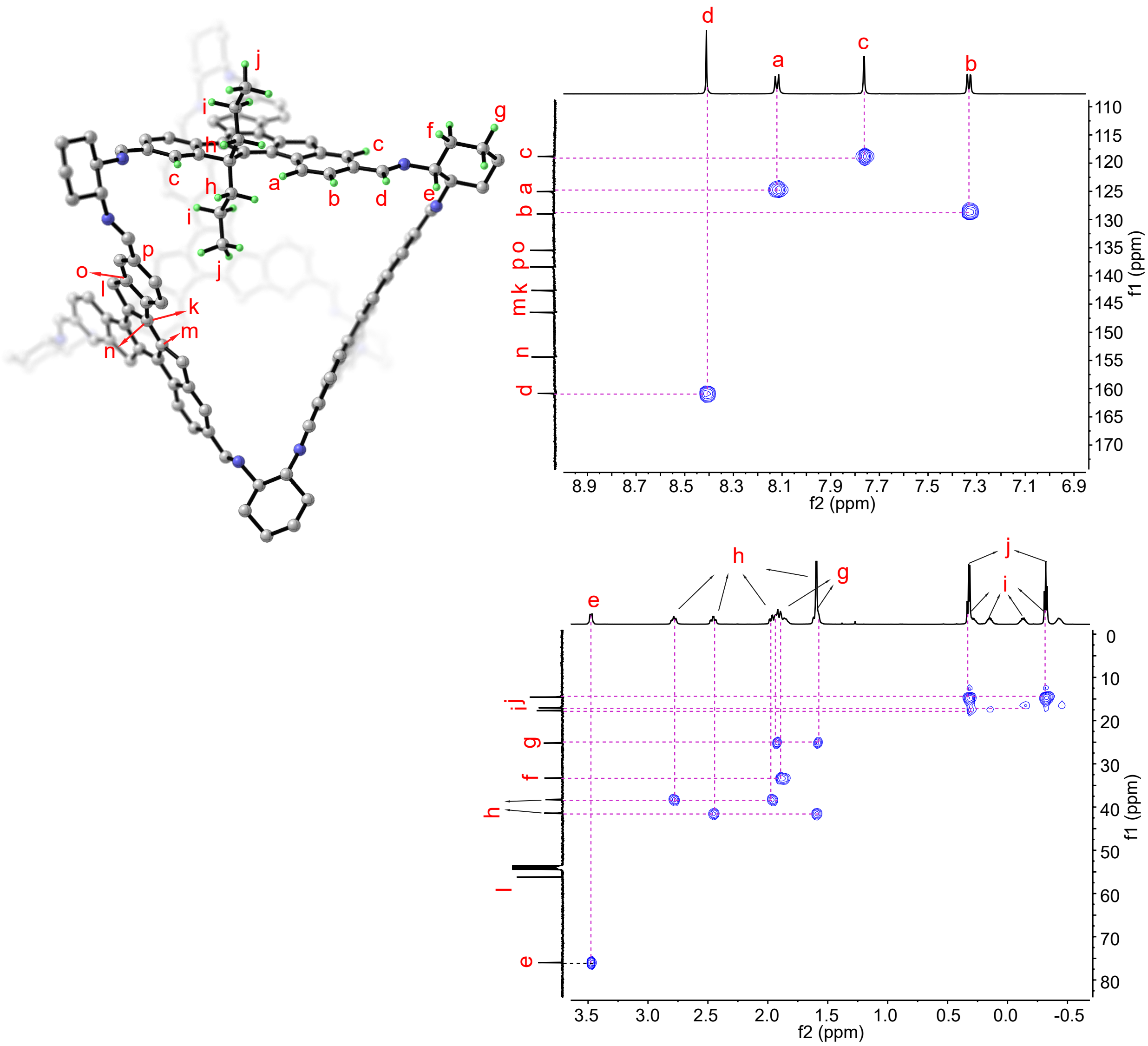

Supplementary Fig. 14. Heteronuclear single-quantum correlation (HSQC) spectrum of 2^6.

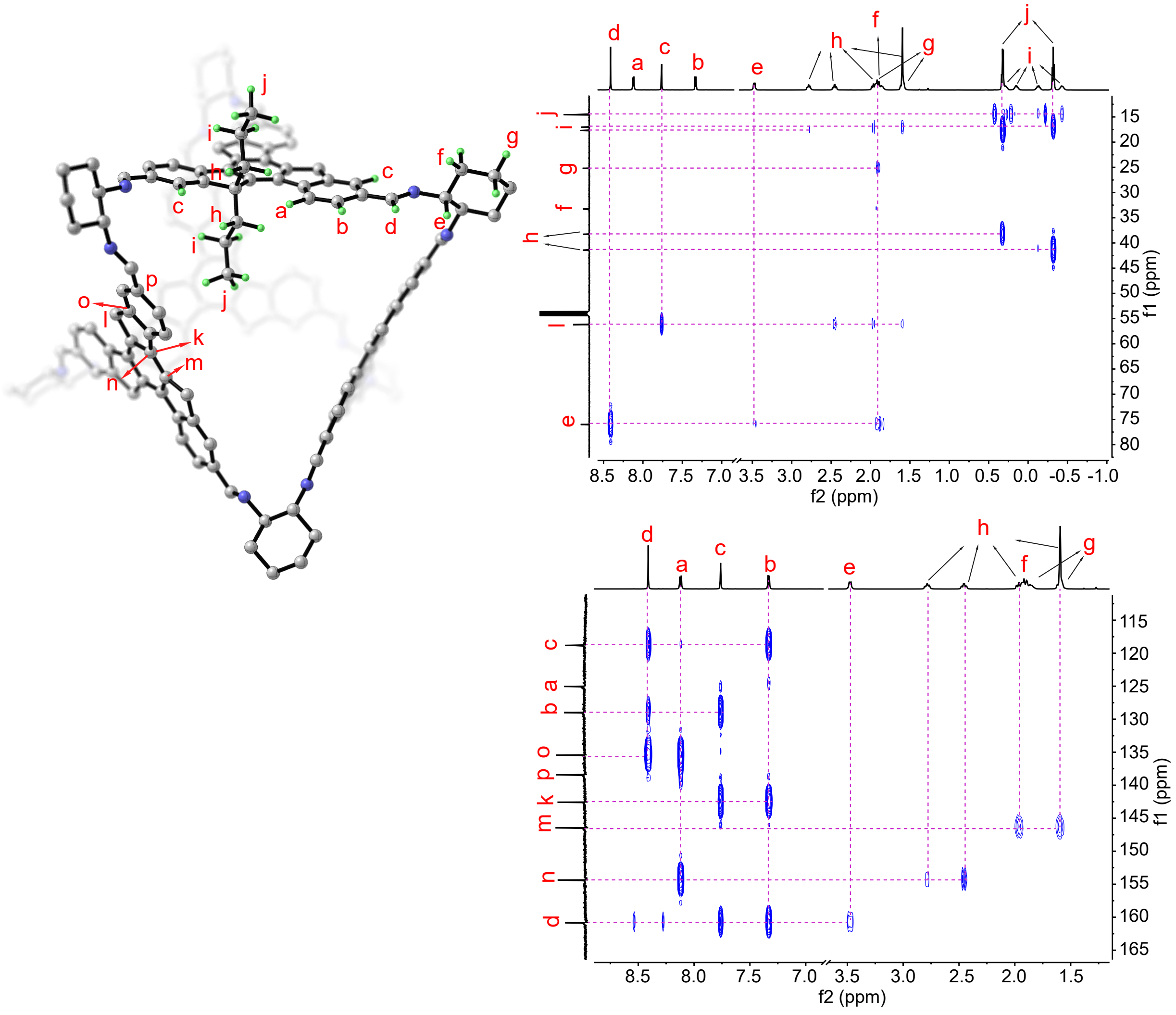

Supplementary Fig. 15. **^{1}H detected heteronuclear multiple bond correlation (HMBC) spectrum of 2^{6}.**

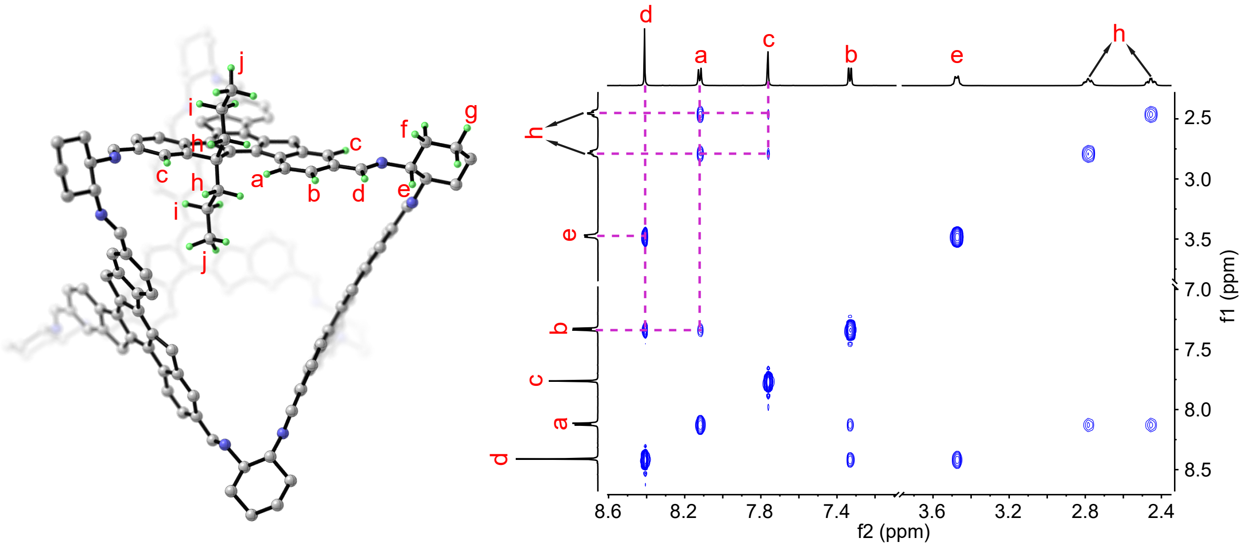

Supplementary Fig. 16. Nuclear Overhauser enhancement spectroscopy (NOESY) spectrum of 2^6.

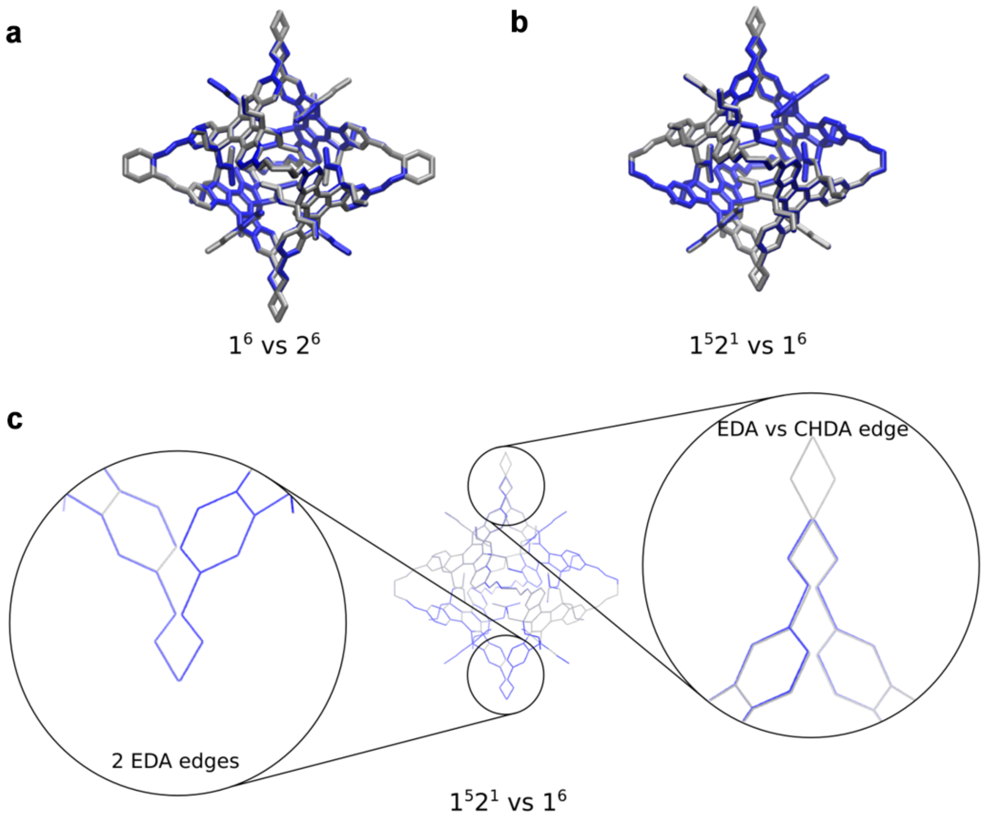

Supplementary Fig. 17. Comparison of DFT optimized structures. (**a**) Aligned optimized structures of $\mathbf{1}^6$ (in blue) and $\mathbf{2}^6$ (in gray). (**b**) Aligned optimized structures of $\mathbf{1}^5\mathbf{2}^1$ (in gray) and $\mathbf{1}^6$ (in blue). (**c**) Same as in part (**b**) but now in line style. The zooms show that only at the vertex where $\mathbf{1}^5\mathbf{2}^1$ has a CHDA and $\mathbf{1}^6$ has an EDA a small difference is present in N-C-C-N dihedral. Because of this also TR is there locally slightly distorted. Structures at all other vertices are close to identical.

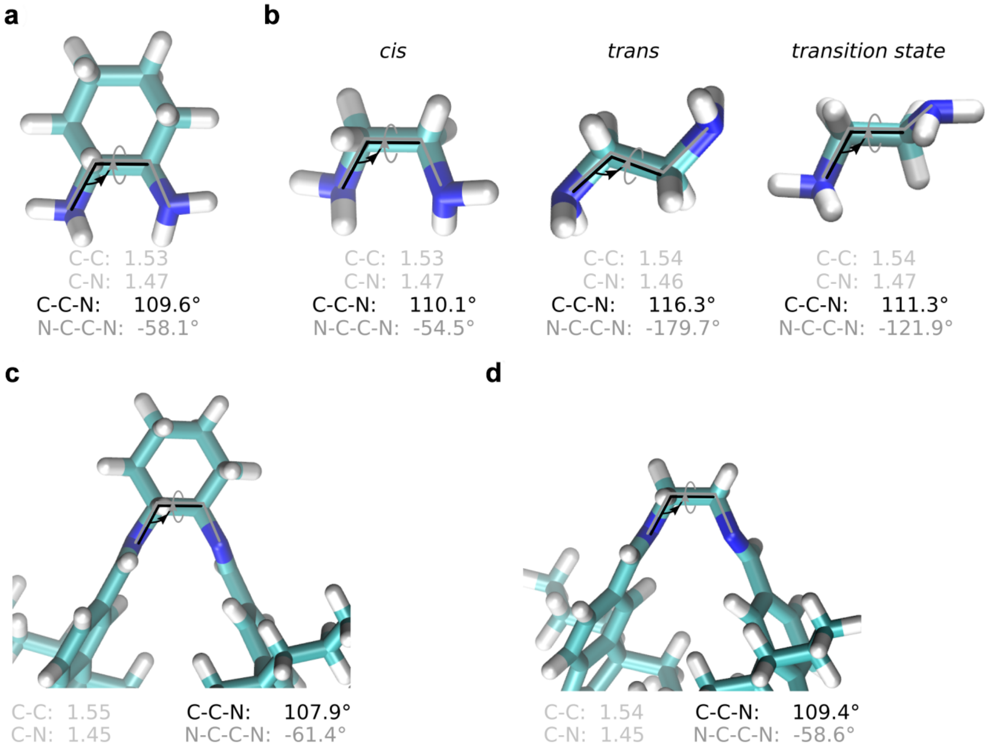

Supplementary Fig. 18. Comparison of bonds, angles and dihedrals in (**a**) CHDA monomer, (**b**) EDA monomer, (**c**) a typical CHDA vertex, and (**d**) a typical EDA vertex as obtained from the DFT optimized structures.

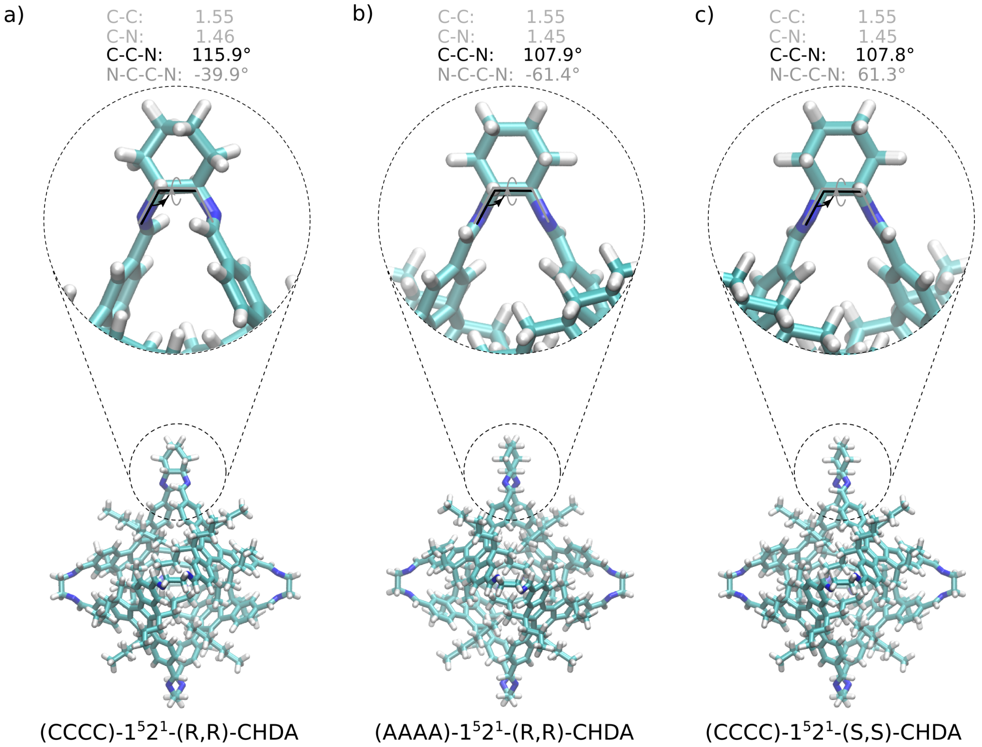

Supplementary Fig. 19. Comparison of DFT optimized structures of (**a**) (*CCCC*)-$\mathbf{1}^5\mathbf{2}^1$-(*R*,*R*)-CHDA, (**b**) (*AAAA*)-$\mathbf{1}^5\mathbf{2}^1$-(*R*,*R*)-CHDA, and (**c**) (*CCCC*)-$\mathbf{1}^5\mathbf{2}^1$-(*S*,*S*)-CHDA. Comparison of (**b**) and (**c**) shows that (*AAAA*)-$\mathbf{1}^5\mathbf{2}^1$-(*R*,*R*)-CHDA, and (*CCCC*)-$\mathbf{1}^5\mathbf{2}^1$-(*S*,*S*)-CHDA are exact mirror images. The calculated energy difference between the two structures is calculated as 0.06 kJ/mol, *i.e.* well within the accuracy of the method. Moreover, comparison of (**a**) and (**b**) shows that the (*R*,*R*)-CHDA has to deform considerably to fit in the *CCCC* structure, resulting in a 71.3 kJ/mol higher energy for the (*CCCC*)-$\mathbf{1}^5\mathbf{2}^1$-(*R*,*R*)-CHDA as compared to the (*AAAA*)-$\mathbf{1}^5\mathbf{2}^1$-(*R*,*R*)-CHDA.

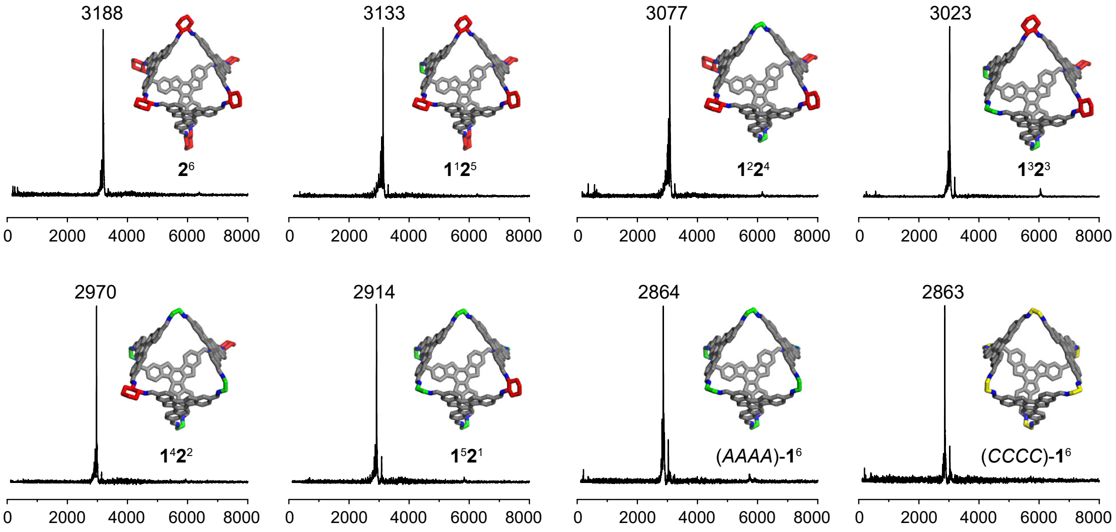

Supplementary Fig. 20. MALDI-TOF mass spectra of the octahedra 1^n2^m separated by HPLC.

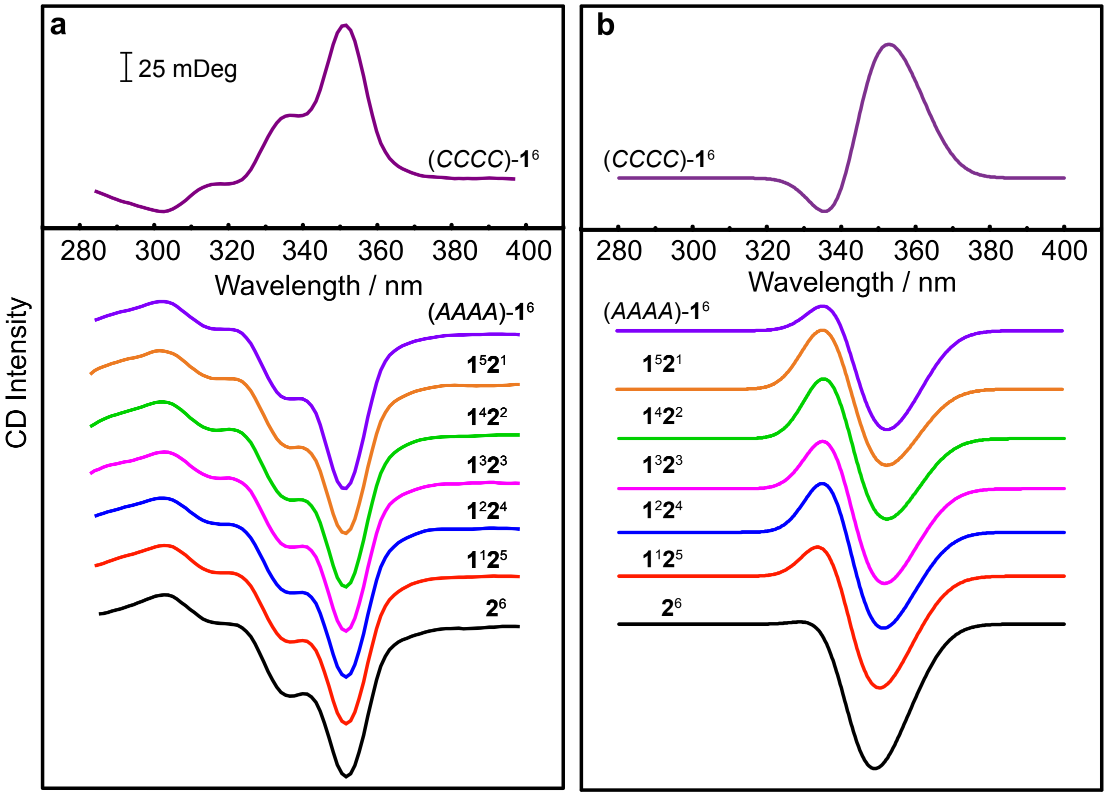

Supplementary Fig. 21. Measured (a) and calculated (b) CD spectra of the octahedra 1^n2^m separated by HPLC.

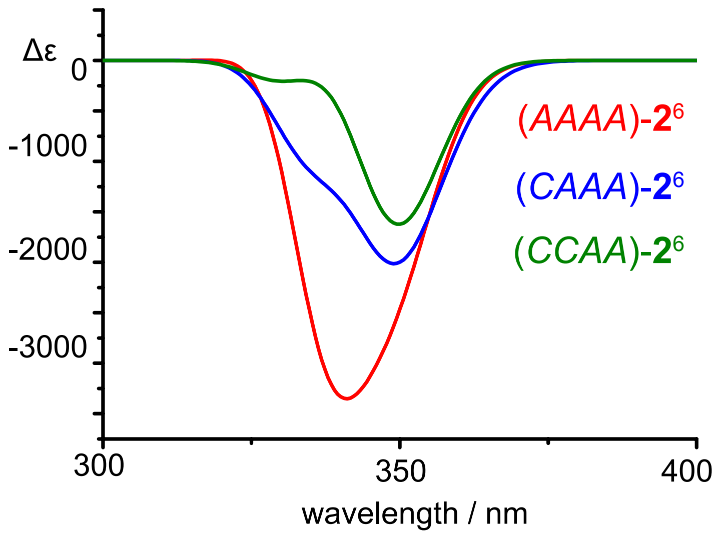

Supplementary Fig. 22. Calculated CD spectra of the octahedra 2^6 with different facial patterns.

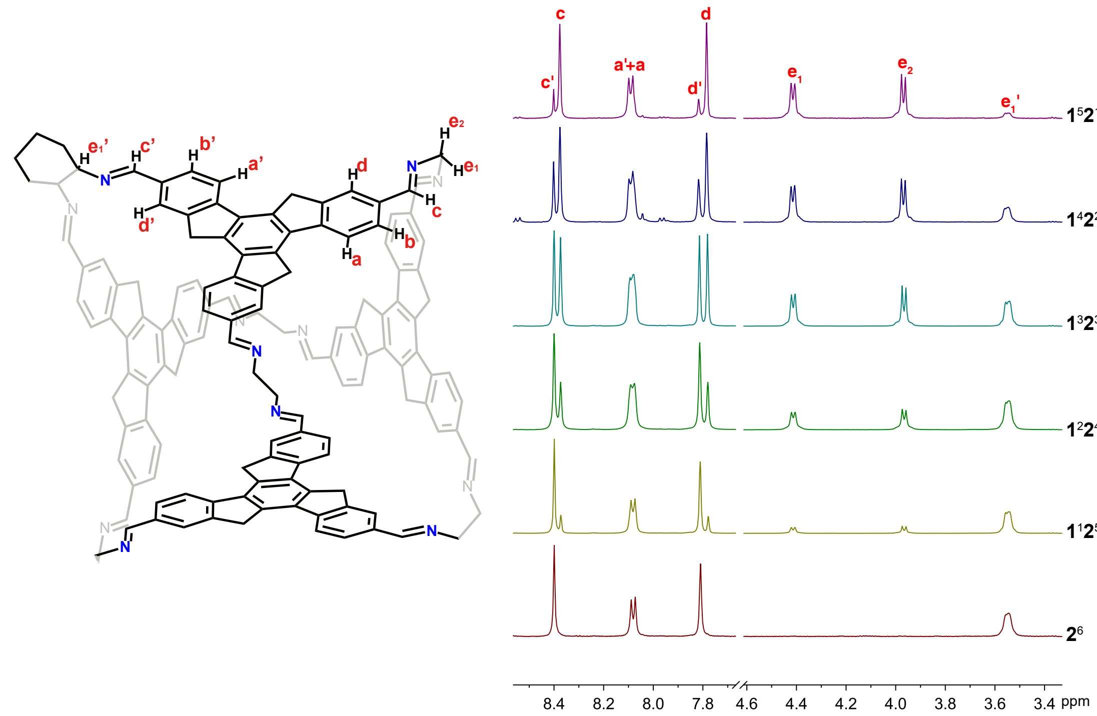

Supplementary Fig. 23. ^{1}H NMR spectra of the octahedra $\mathbf{1}^n\mathbf{2}^m$ separated by HPLC. The n and m values of each octahedra $\mathbf{1}^n\mathbf{2}^m$ completely matched the integrations of the protons nearby the EDA-linked vertex (H^c and H^d) and the CHDA-linked vertex ($H^{c'}$ and $H^{d'}$).

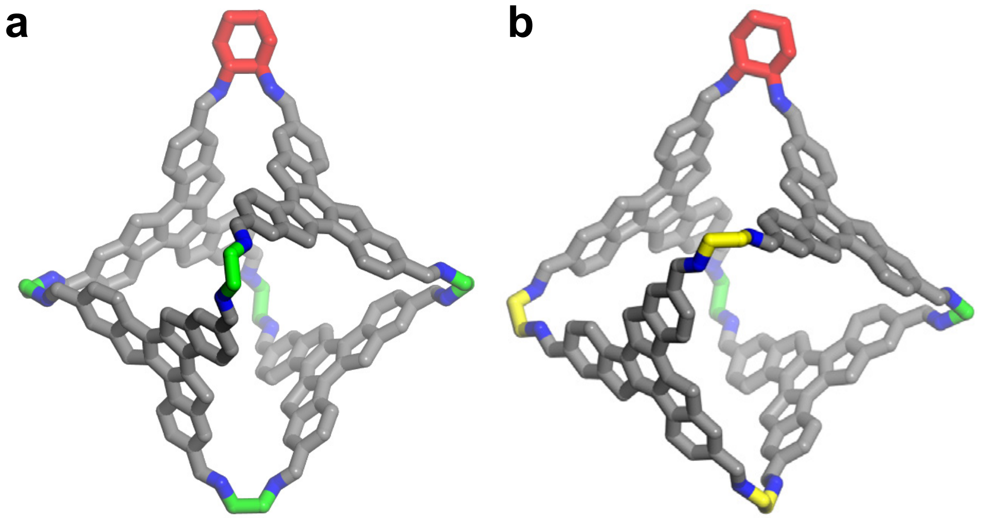

Supplementary Fig. 24. The structures of (*CCCC*)-$\mathbf{1}^5\mathbf{2}^1$ with all EDA-linked vertices in *c.a.* $-60°$ gauche conformation (**a**), and with three EDA-linked vertices in *c.a.* $60°$ gauche conformation (**b**) optimized by COMPASS II force field and VASP. The red vertex presents (*R*,*R*)-CHDA-linked vertex; the yellow and green vertices present EDA-linked vertices in the gauche conformations with dihedral angles of *c.a.* $60°$ and *c.a.* $-60°$ respectively. The free energies of these two structures are given in Supplementary Table 4.

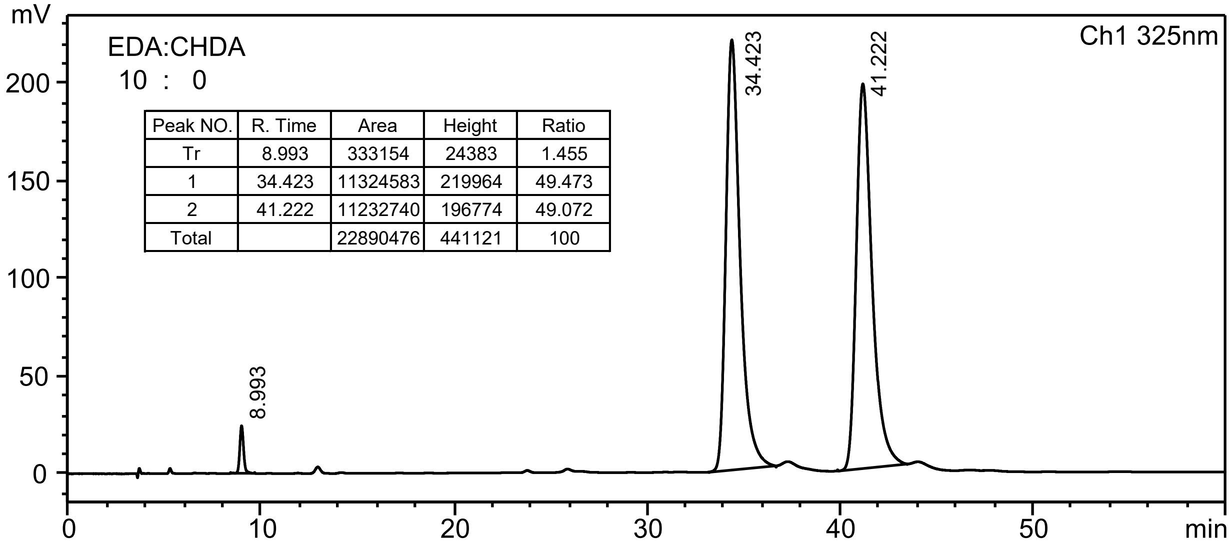

Peak NO.	R. Time	Area	Height	Ratio
Tr	8.993	333154	24383	1.455
1	34.423	11324583	219964	49.473
2	41.222	11232740	196774	49.072
Total		22890476	441121	100

Supplementary Fig. 25. HPLC spectrum of the equilibrium product containing 0% CHDA.

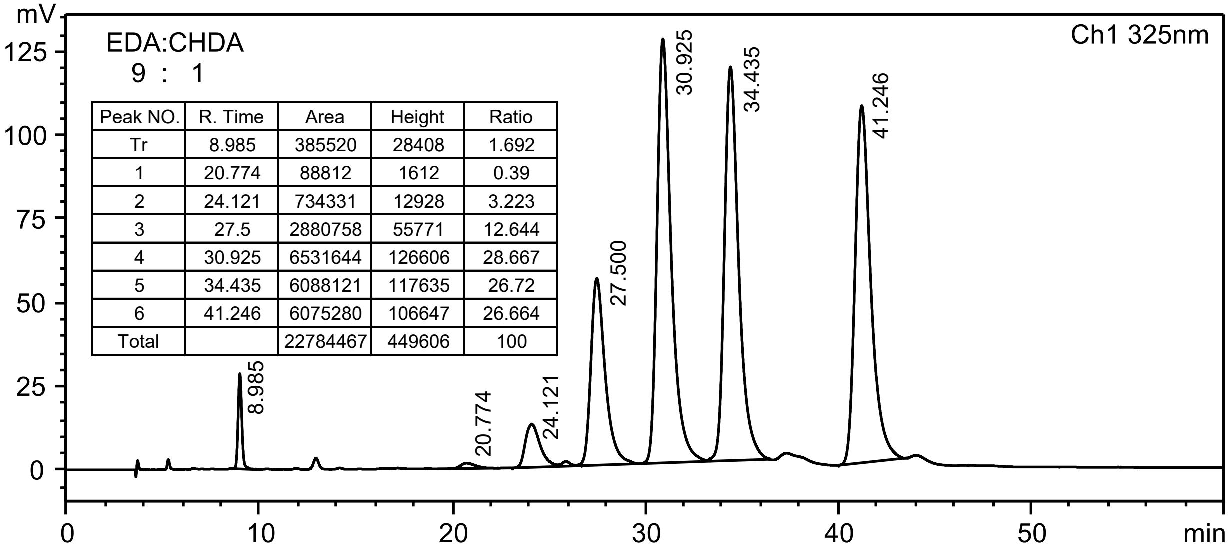

Peak NO.	R. Time	Area	Height	Ratio
Tr	8.985	385520	28408	1.692
1	20.774	88812	1612	0.39
2	24.121	734331	12928	3.223
3	27.5	2880758	55771	12.644
4	30.925	6531644	126606	28.667
5	34.435	6088121	117635	26.72
6	41.246	6075280	106647	26.664
Total		22784467	449606	100

Supplementary Fig. 26. HPLC spectrum of the equilibrium product containing 10% CHDA.

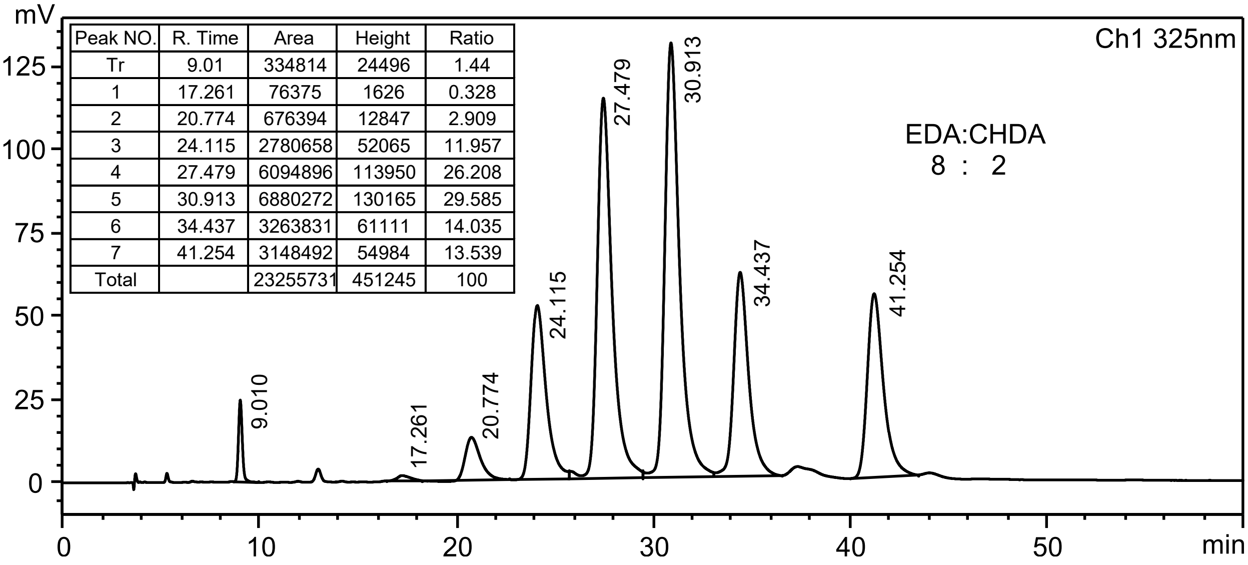

Peak NO.	R. Time	Area	Height	Ratio
Tr	9.01	334814	24496	1.44
1	17.261	76375	1626	0.328
2	20.774	676394	12847	2.909
3	24.115	2780658	52065	11.957
4	27.479	6094896	113950	26.208
5	30.913	6880272	130165	29.585
6	34.437	3263831	61111	14.035
7	41.254	3148492	54984	13.539
Total		23255731	451245	100

Supplementary Fig. 27. HPLC spectrum of the equilibrium product containing 20% CHDA.

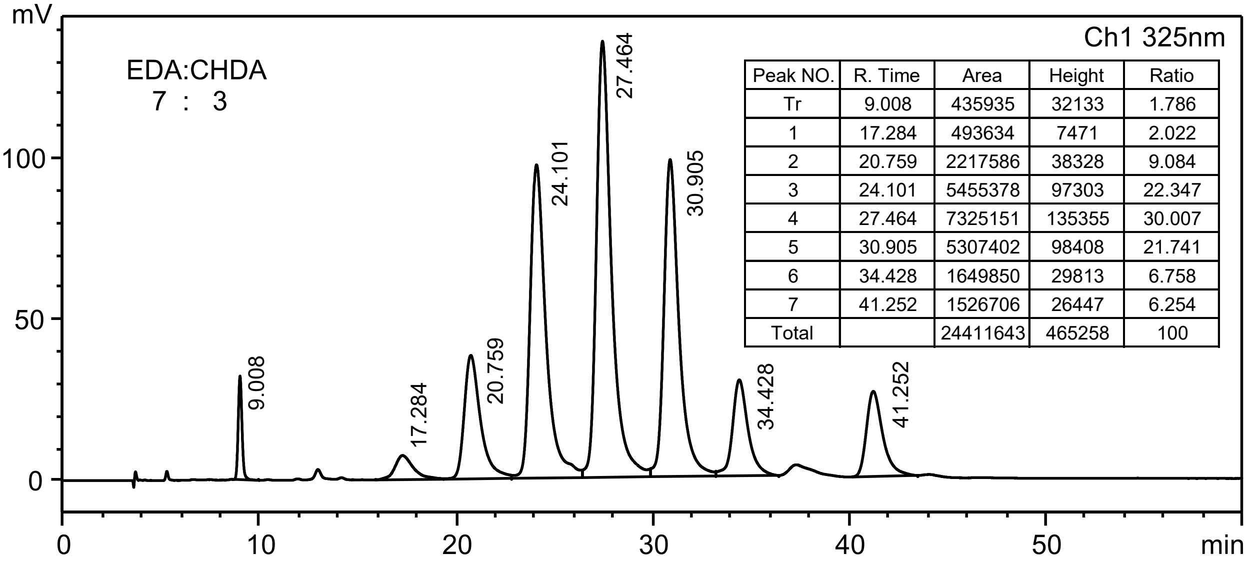

Peak NO.	R. Time	Area	Height	Ratio
Tr	9.008	435935	32133	1.786
1	17.284	493634	7471	2.022
2	20.759	2217586	38328	9.084
3	24.101	5455378	97303	22.347
4	27.464	7325151	135355	30.007
5	30.905	5307402	98408	21.741
6	34.428	1649850	29813	6.758
7	41.252	1526706	26447	6.254
Total		24411643	465258	100

Supplementary Fig. 28. HPLC spectrum of the equilibrium product containing 30% CHDA.

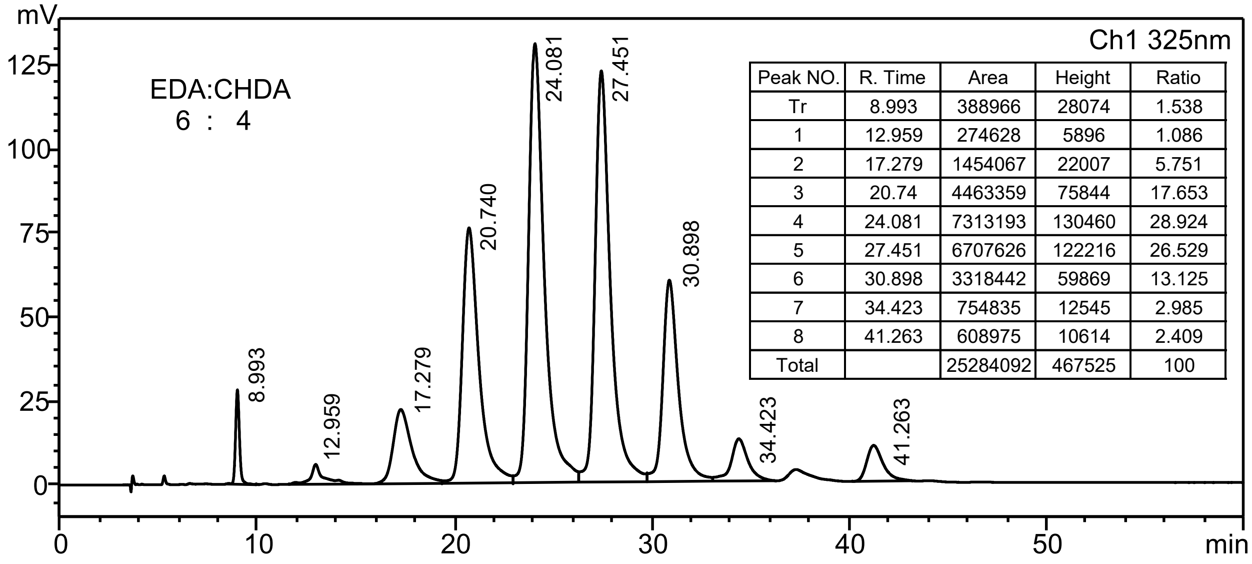

Peak NO.	R. Time	Area	Height	Ratio
Tr	8.993	388966	28074	1.538
1	12.959	274628	5896	1.086
2	17.279	1454067	22007	5.751
3	20.74	4463359	75844	17.653
4	24.081	7313193	130460	28.924
5	27.451	6707626	122216	26.529
6	30.898	3318442	59869	13.125
7	34.423	754835	12545	2.985
8	41.263	608975	10614	2.409
Total		25284092	467525	100

Supplementary Fig. 29. HPLC spectrum of the equilibrium product containing 40% CHDA.

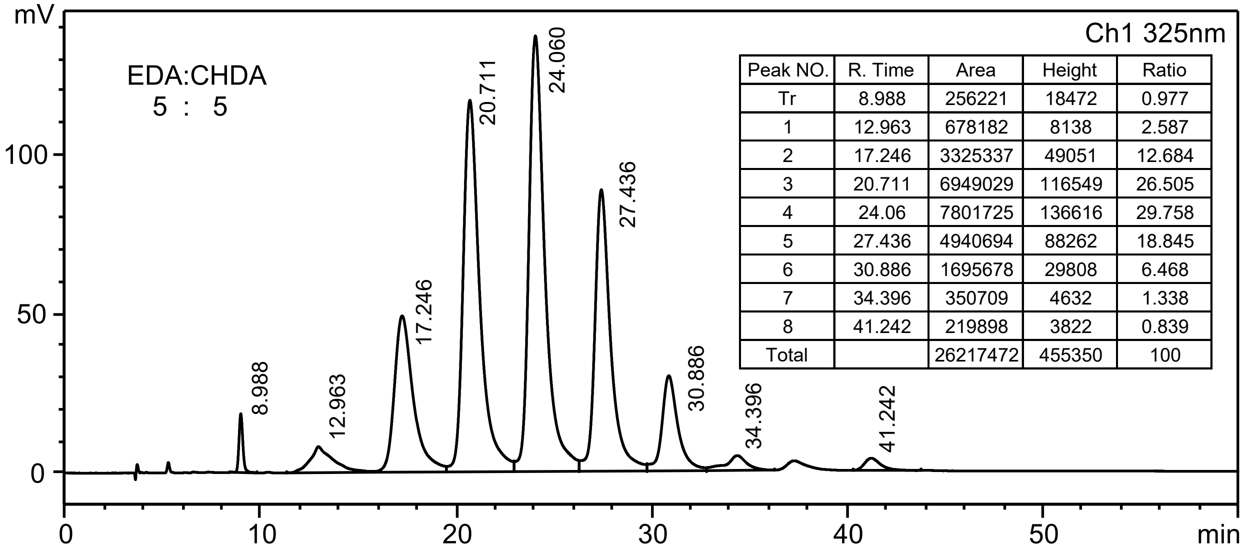

Peak NO.	R. Time	Area	Height	Ratio
Tr	8.988	256221	18472	0.977
1	12.963	678182	8138	2.587
2	17.246	3325337	49051	12.684
3	20.711	6949029	116549	26.505
4	24.06	7801725	136616	29.758
5	27.436	4940694	88262	18.845
6	30.886	1695678	29808	6.468
7	34.396	350709	4632	1.338
8	41.242	219898	3822	0.839
Total		26217472	455350	100

Supplementary Fig. 30. HPLC spectrum of the equilibrium product containing 50% CHDA.

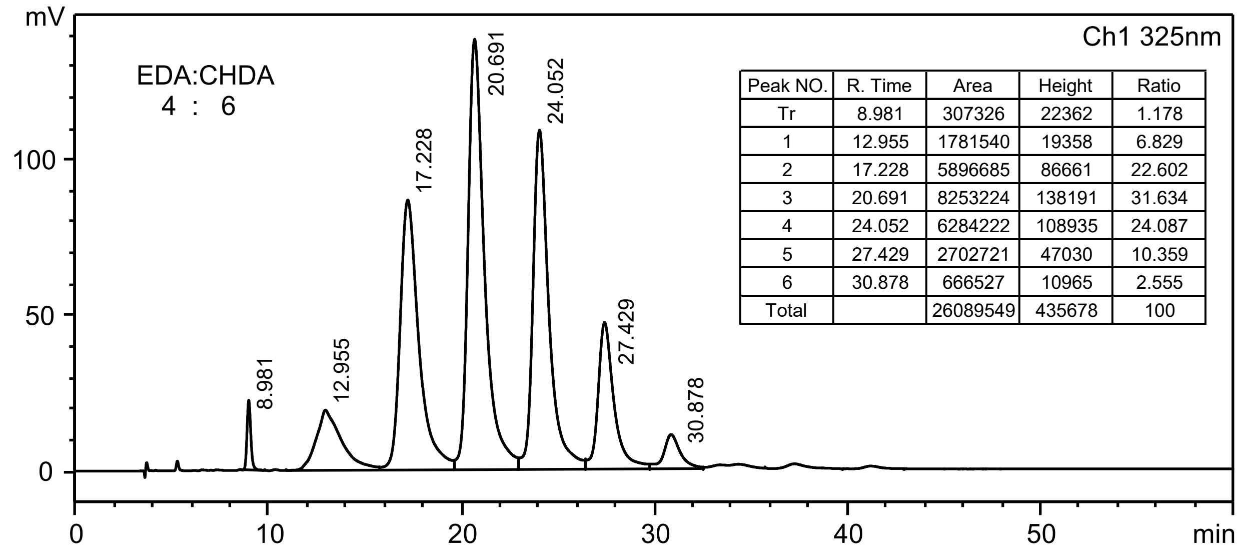

Peak NO.	R. Time	Area	Height	Ratio
Tr	8.981	307326	22362	1.178
1	12.955	1781540	19358	6.829
2	17.228	5896685	86661	22.602
3	20.691	8253224	138191	31.634
4	24.052	6284222	108935	24.087
5	27.429	2702721	47030	10.359
6	30.878	666527	10965	2.555
Total		26089549	435678	100

Supplementary Fig. 31. HPLC spectrum of the equilibrium product containing 60% CHDA.

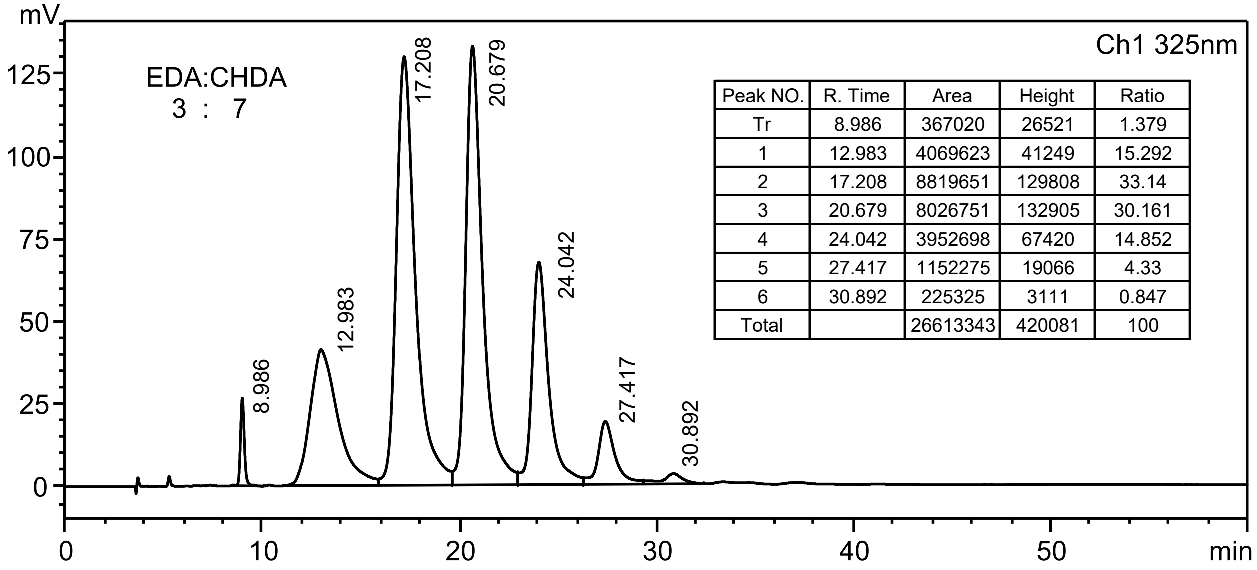

Peak NO.	R. Time	Area	Height	Ratio
Tr	8.986	367020	26521	1.379
1	12.983	4069623	41249	15.292
2	17.208	8819651	129808	33.14
3	20.679	8026751	132905	30.161
4	24.042	3952698	67420	14.852
5	27.417	1152275	19066	4.33
6	30.892	225325	3111	0.847
Total		26613343	420081	100

Supplementary Fig. 32. HPLC spectrum of the equilibrium product containing 70% CHDA.

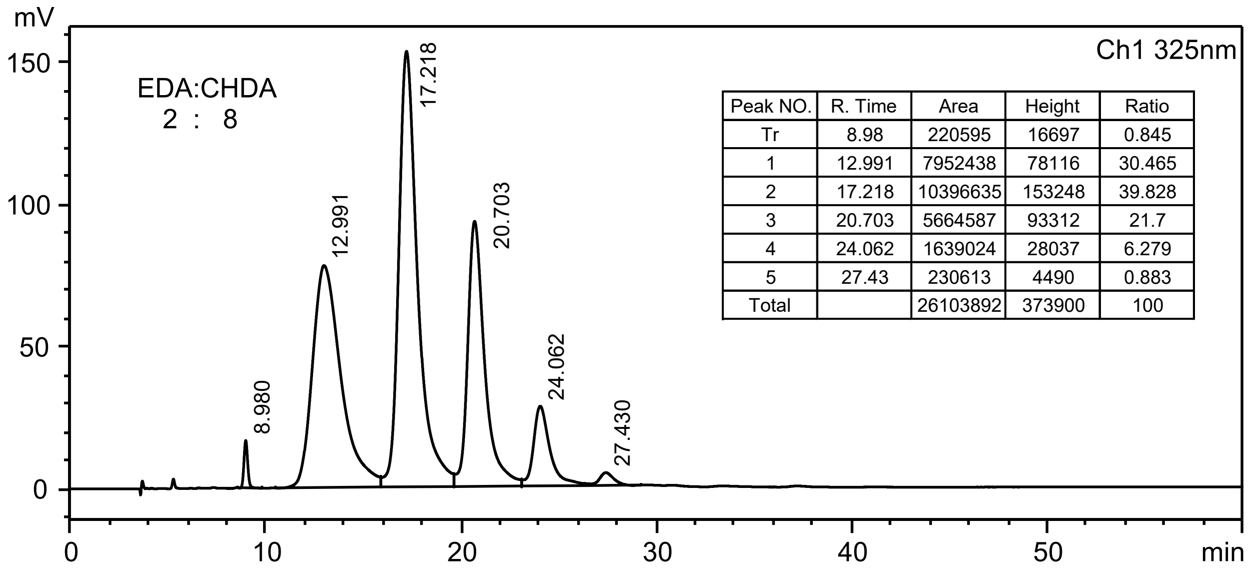

Peak NO.	R. Time	Area	Height	Ratio
Tr	8.98	220595	16697	0.845
1	12.991	7952438	78116	30.465
2	17.218	10396635	153248	39.828
3	20.703	5664587	93312	21.7
4	24.062	1639024	28037	6.279
5	27.43	230613	4490	0.883
Total		26103892	373900	100

Supplementary Fig. 33. HPLC spectrum of the equilibrium product containing 80% CHDA.

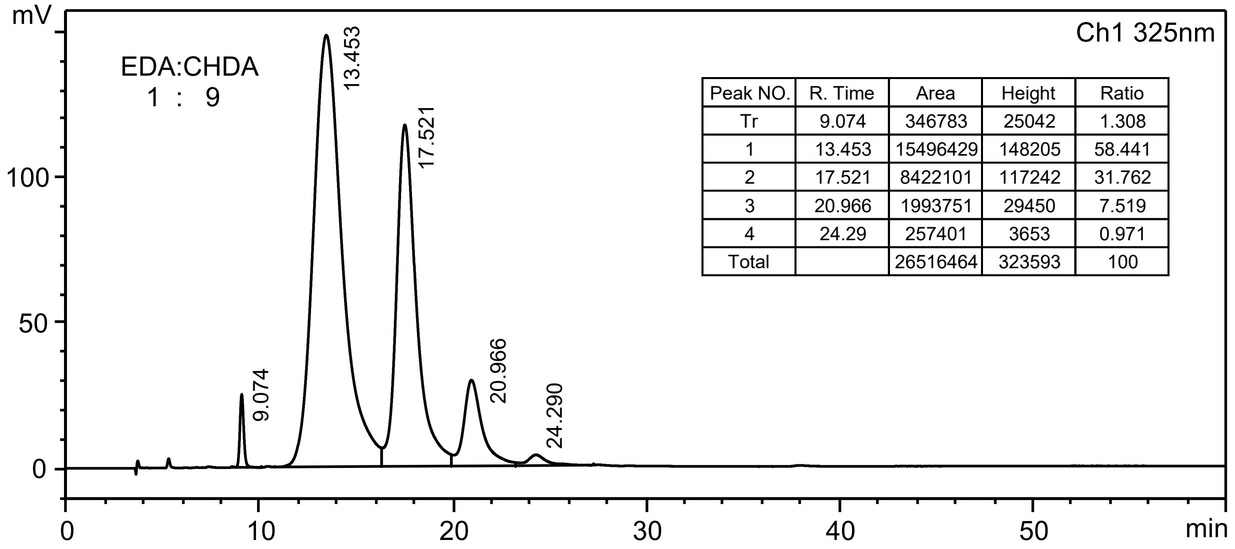

Peak NO.	R. Time	Area	Height	Ratio
Tr	9.074	346783	25042	1.308
1	13.453	15496429	148205	58.441
2	17.521	8422101	117242	31.762
3	20.966	1993751	29450	7.519
4	24.29	257401	3653	0.971
Total		26516464	323593	100

Supplementary Fig. 34. HPLC spectrum of the equilibrium product containing 90% CHDA.

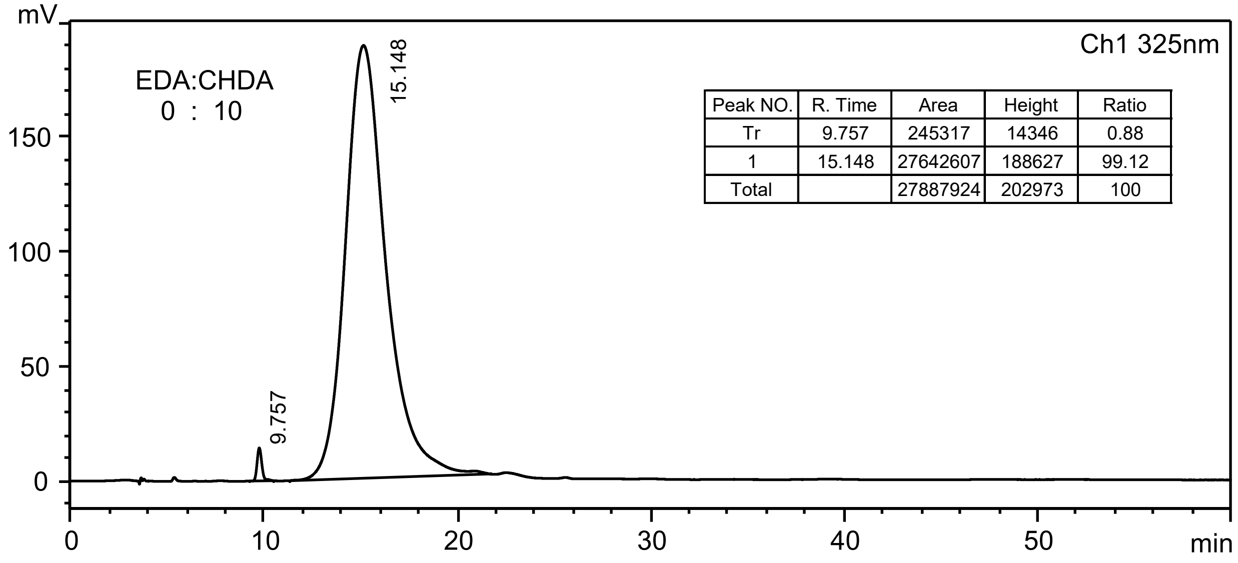

Peak NO.	R. Time	Area	Height	Ratio
Tr	9.757	245317	14346	0.88
1	15.148	27642607	188627	99.12
Total		27887924	202973	100

Supplementary Fig. 35. HPLC spectrum of the equilibrium product containing 100% CHDA.

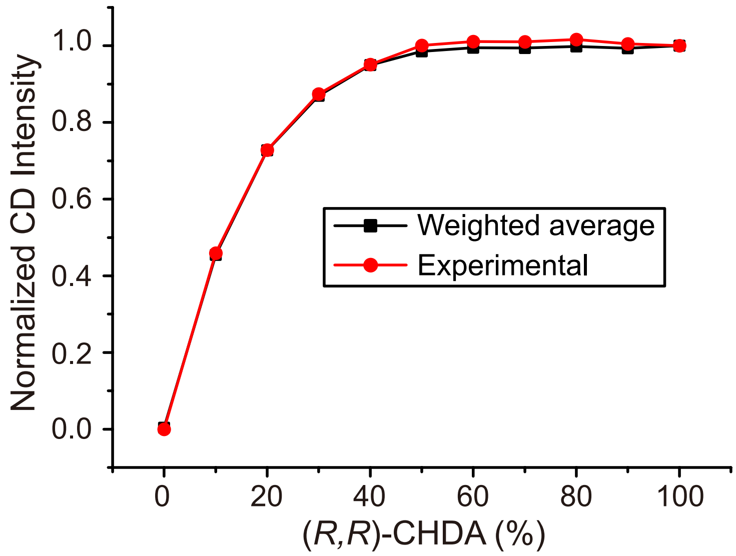

Supplementary Fig. 36. Weighted averaged CD intensity (black) was calculated by multiplying the CD intensities of each octahedron by their fractions in the equilibrium product, whereas the experimental normalized CD intensity (red) was directly measured from the equilibrium product mixtures. The consistency of these two results illustrates that the general "sergeants-and-soldiers" effect in a "company" is a weighted average of the effects in each "squad".

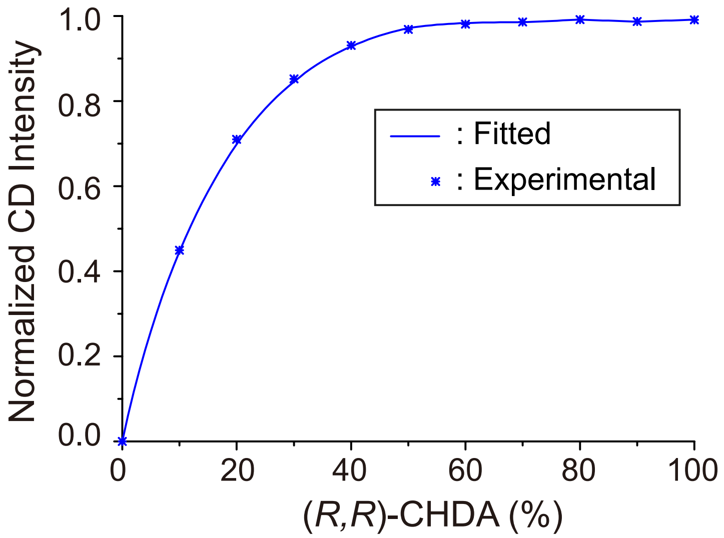

Supplementary Fig. 37. Fitted (line) and experimental (points) CD intensities of the equilibrium products with various ratios of CHDA. Fitted results are obtained from the theoretical model based on the mass balance approach, whereas the experimental results are measured directly.

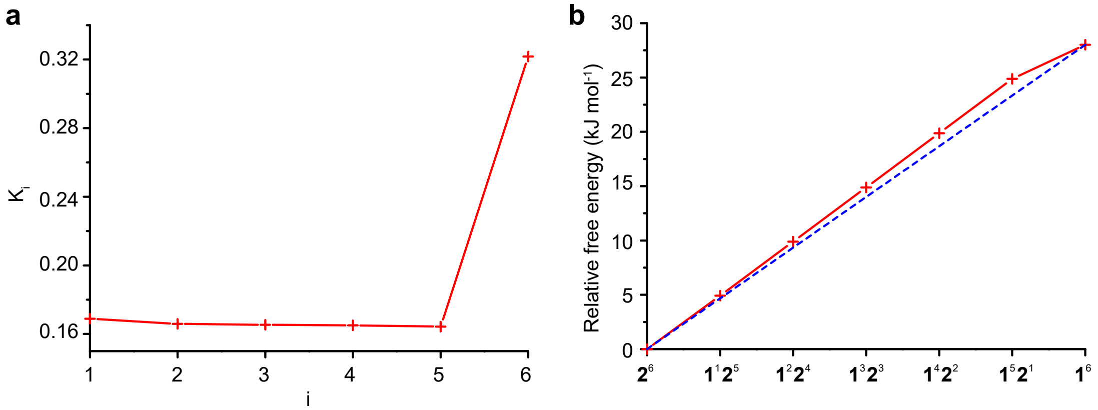

Supplementary Fig. 38. Fitted equilibrium constants K_i $(1 \leq i \leq 6)$ (**a**) and corresponding relative free energies (**b**) for the different types of octahedra. The blue line indicates the case that all equilibrium constants (and thus free energy differences between adjacent octahedra types) would be equal. The figure illustrates that the free energy of the octahedra decreases with the amount of CHDA-linked vertices inside.

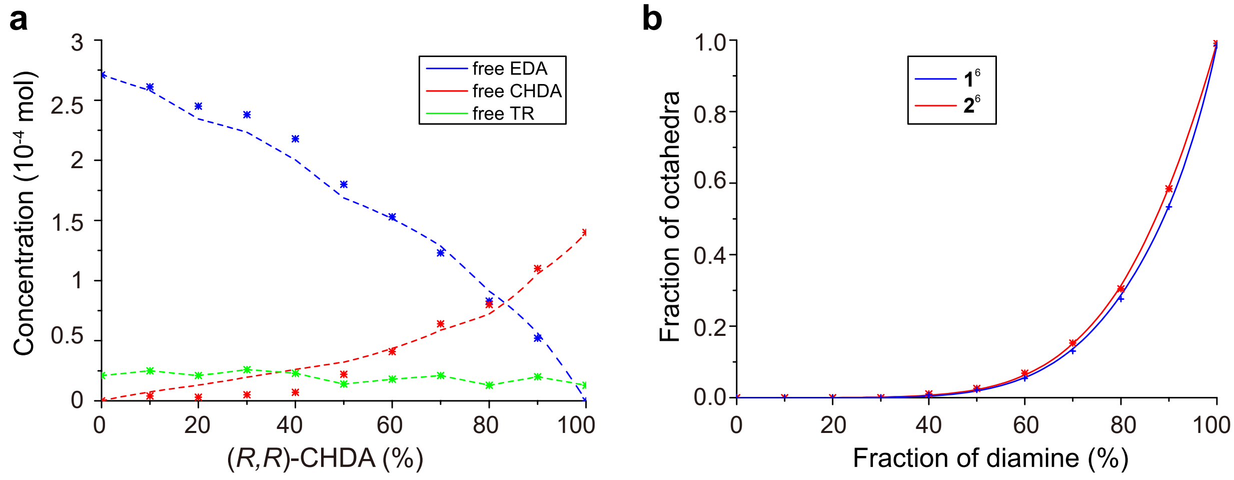

Supplementary Fig. 39. (**a**) Fitted (dashed lines) and experimental (star points) concentrations of free EDA, CHDA and TR. (**b**) Y-axis refers to fitted (lines) and experimental (points) fractions of the octahedra with exclusive diamine vertex (*i.e.*, $\mathbf{1}^6$ and $\mathbf{2}^6$), while the x-axis differs for the red and the blue curve. For the red curve it is the overall fraction of CHDA, for the blue the overall fraction of EDA. For a same excess of major diamine vertex, the blue line is always below the red line, thus illustrating that $\mathbf{2}^6$ should have lower free energy then $\mathbf{1}^6$. Fitted results are obtained from the theoretical mass balance model based on the overall concentrations EDA, CHDA and TR as well as the experimentally observed free TR concentrations, whereas the experimental results are obtained from the calculation in Supplementary Table 3.

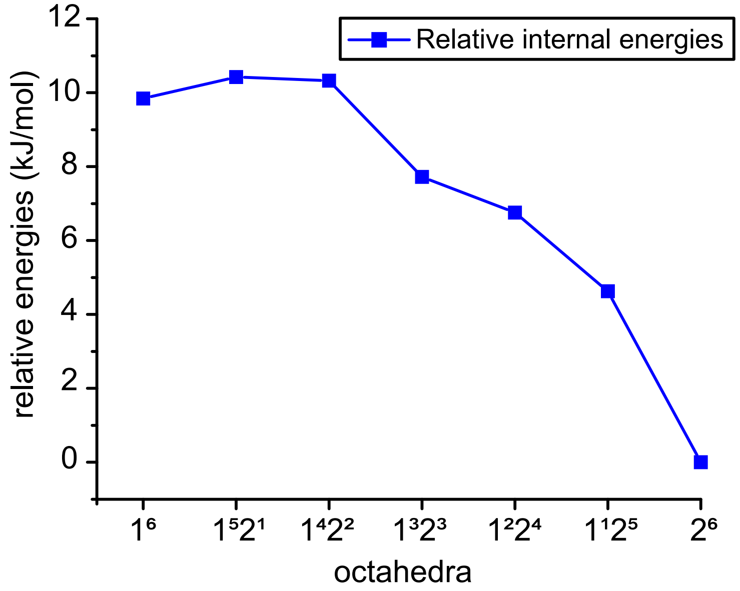

Supplementary Fig. 40. Relative internal energies upon exchange of CHDA and EDA vertices, as calculated from the data in Supplementary Table 4.

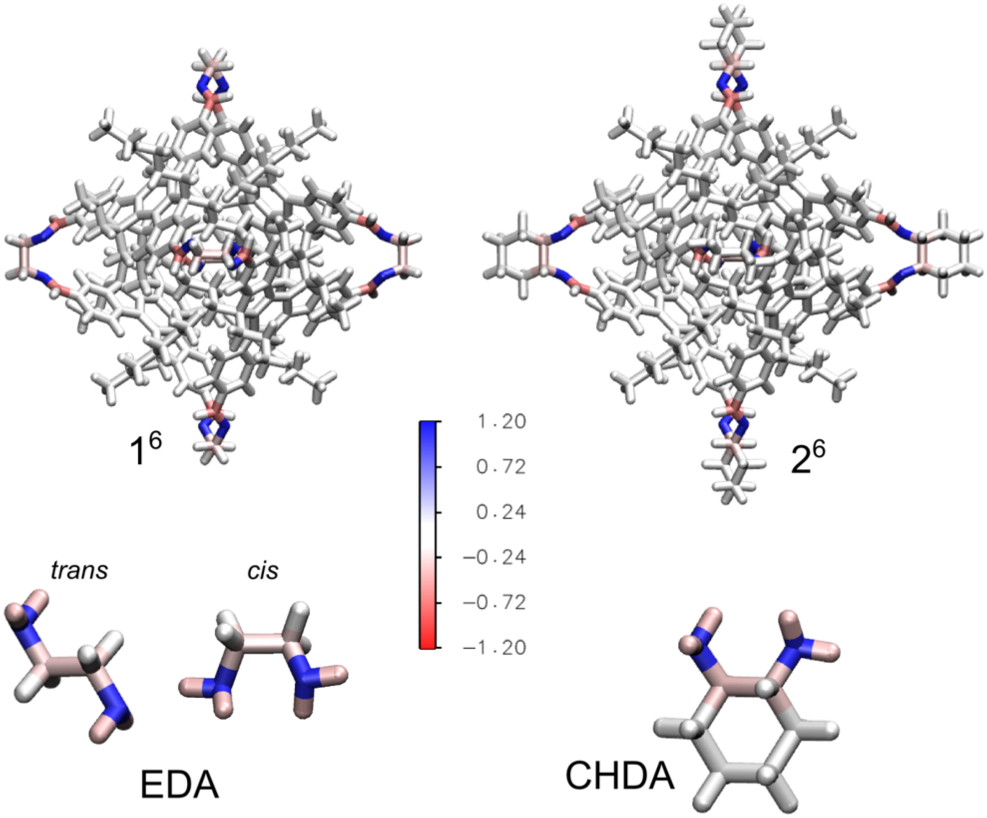

Supplementary Fig. 41. Excess electric charge per atom (*i.e.*, q-1 for H, q-4 for C, and q-5 for N) in the structures of $\mathbf{1}^6$, $\mathbf{2}^6$, EDA monomer and CHDA monomer as calculated using DFT, where the color indicates the excess electronic charge on each atom. Average number of electrons per atom for atoms at the vertices is shown in Supplementary Table 6.

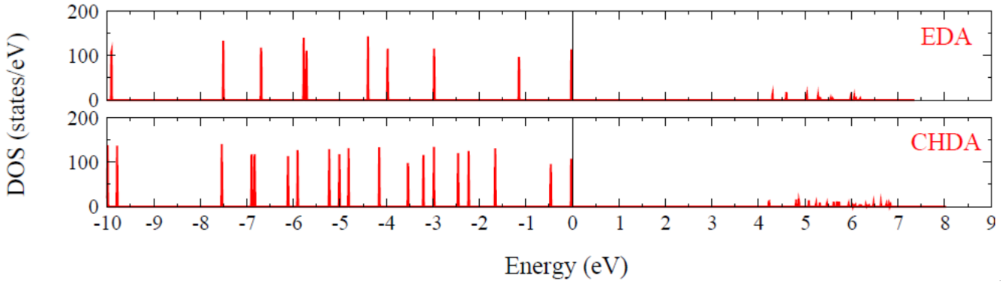

Supplementary Fig. 42. Density of states for free EDA and CHDA monomers. The HOMO-level has been set to zero. CHDA has states closer to the Fermi-level, indicating that it is more reactive than EDA.

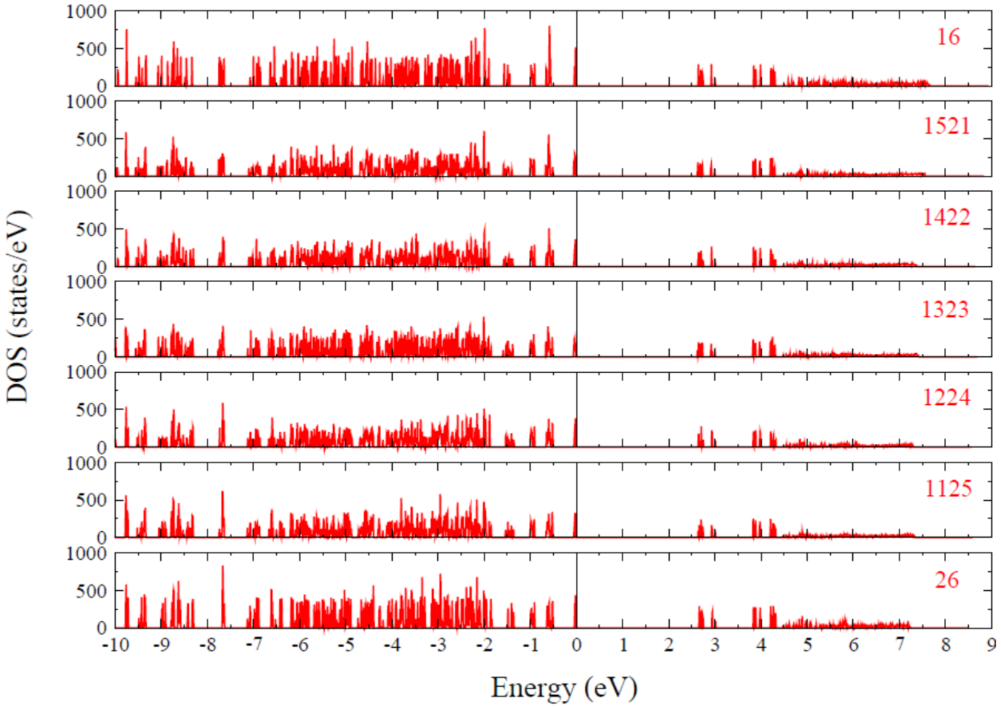

Supplementary Fig. 43. Density of states for the 7 different octahedra. The HOMO-level has been set to zero. Only minor differences are observed between the octahedra with different ratios of EDA-linked and CHDA-linked vertices.

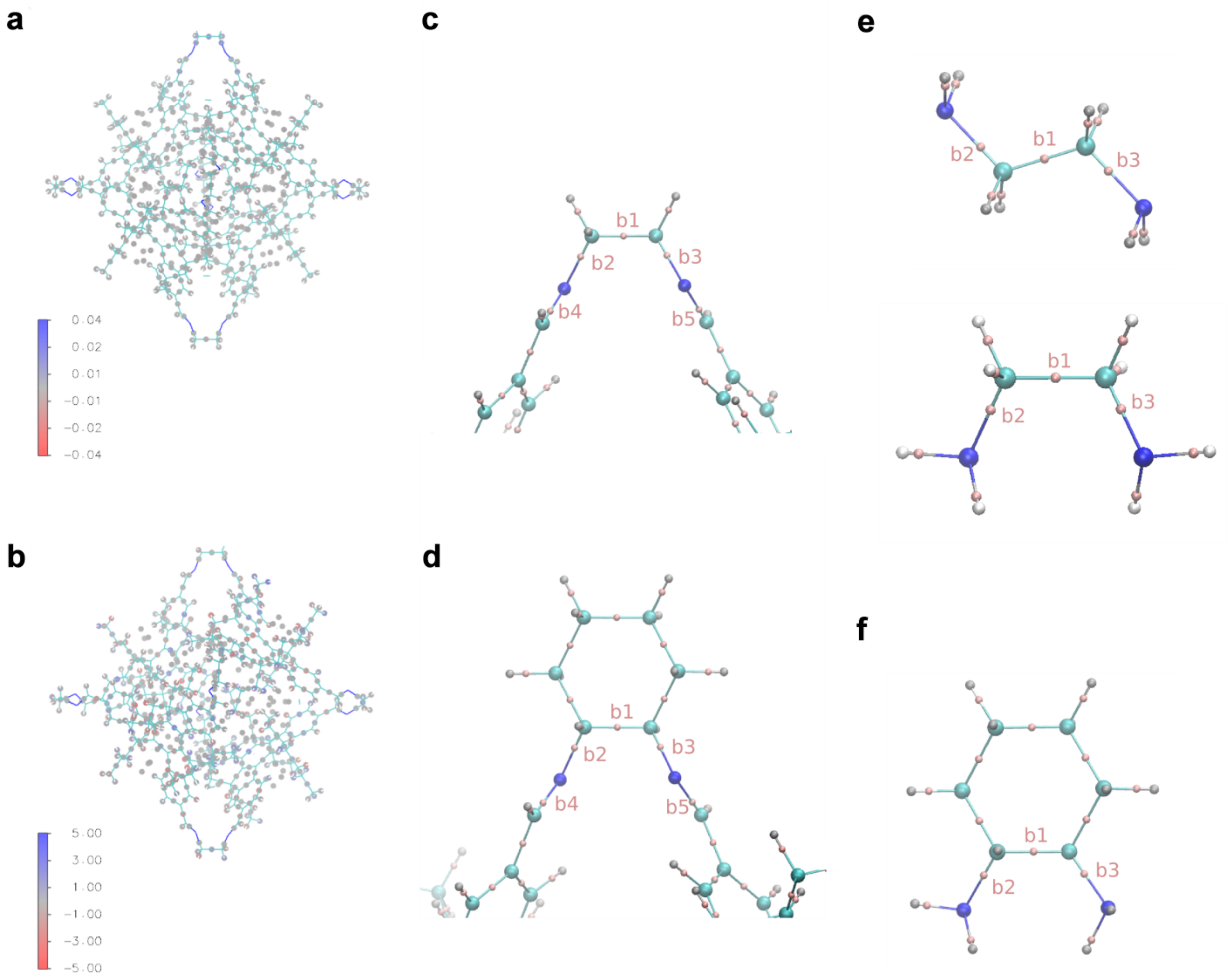

Supplementary Fig. 44. Bond critical points as calculated with DFT: (**a**) Structure of $\mathbf{1}^5\mathbf{2}^1$ in lines and bond critical points (for bonds that are present in $\mathbf{1}^5\mathbf{2}^1$ as well as in $\mathbf{1}^6$), shown as points where the color indicates the difference in electronic charge between $\mathbf{1}^5\mathbf{2}^1$ and $\mathbf{1}^6$. Shows that there is hardly any difference, with largest difference at the vertex where an EDA is replaced by a CHDA. (**b**) idem, but now color of points indicates difference in Laplacian. (**c**) Zoom in on one of the EDA vertices of $\mathbf{1}^5\mathbf{2}^1$, with the critical points shown as light red spheres. Values for the electronic charge and Laplacian are given in Supplementary Table 7. (**d**) as in part (**c**) but now for the CHDA vertex of $\mathbf{1}^5\mathbf{2}^1$. (**e**) EDA monomer with the critical points shown as light red spheres, and (**f**) CHDA monomer with the critical points shown as light red spheres.

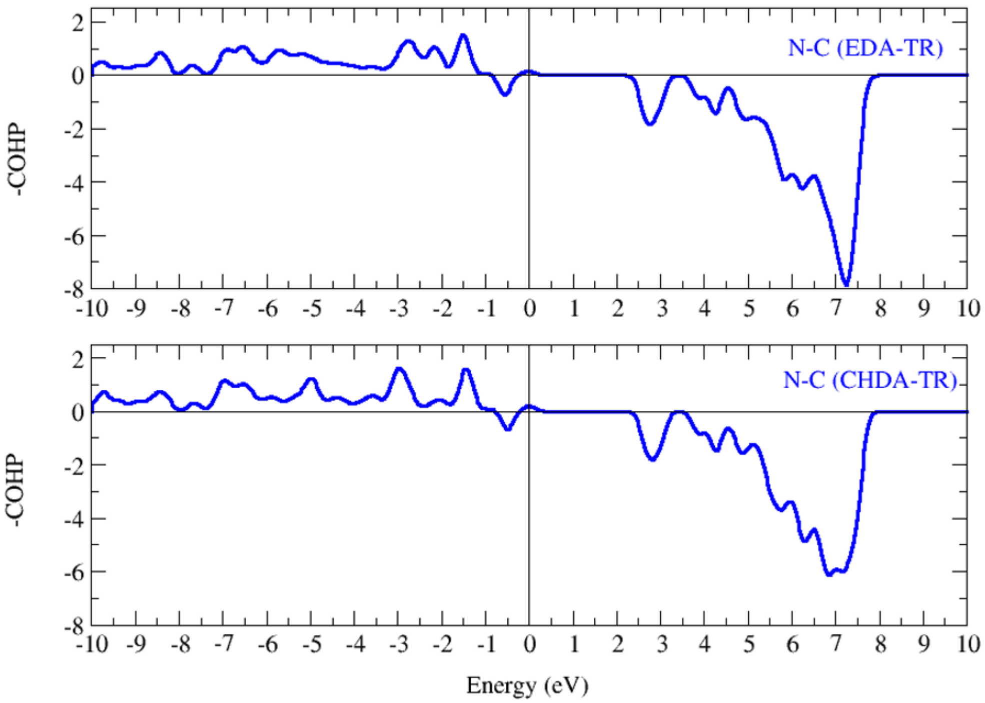

Supplementary Fig. 45. COHP plots for the N-C bond of EDA-TR and CHDA-TR. In both cases the bonding domain is fully occupied, whereas small domains of anti-bonding are also filled.

Supplementary Tables

Supplementary Table 1. Crystallographic data of **1**[6]

Compound	**1**[6]
Empirical formula	C_{204} H_{240} N_{12}
Formula weight	2860.07
Temperature / K	100
Crystal system	cubic
Space group	*P*-43*n*
a / Å	36.41557(19)
b / Å	36.41557(19)
c / Å	36.41557(19)
α / °	90
β / °	90
γ / °	90
Volume / Å^3	48290.5(8)
Z	8
ρ_{calc} / g cm^{-3}	0.787
μ / mm^{-1}	0.341
F(000)	12384
Crystal size / mm^3	0.4 × 0.3 × 0.3
Radiation	CuKα(λ=1.54178)
2Θ range for data collection / °	5.944 to 141.590
Index ranges	-44≥h≥43, -44≥k≥29, -44≥l≥43
Reflections collected	317664
Independent reflections	15450[R_{int}=0.0804]
Data/restraints/parameters	15450/19/659
Goodness-of-fit on F^2	1.058
Final R indexes [I≥2σ(I)]	R_1=0.0588, wR_2=0.1791
Final R indexes [all data]	R_1=0.0704, wR_2=0.1955
Largest diff. peak/hole /e Å^{-3}	0.33/-0.14
Flack parameter	0.38(18)
CCDC[#]	1517934

Crystallographic data have been submitted to the Cambridge Crystallographic Database with according reference numbers and are available free of charge at http://www.ccdc.cam.ac.uk/data_request/cif.

Supplementary Table 2. Construction of the dynamic libraries containing designed ratios of TR, EDA, and CHDA.

Sample	v(TR) /μL	v(EDA) /μL	v(CHDA) /μL	v(TFA) /μL	v(Tol) /μL	$[TR]_0$ /mM	$[EDA]_0$ /mM	$[CHDA]_0$ /mM	$[EDA]_0$ %	$[CHDA]_0$ %
A	900	500	0	50	550	1.440	2.40	0	100	0
B	905	450	50	50	545	1.448	2.16	0.24	90	10
C	910	400	100	50	540	1.456	1.92	0.48	80	20
D	915	350	150	50	535	1.464	1.68	0.72	70	30
E	920	300	200	50	530	1.472	1.44	0.96	60	40
F	925	250	250	50	525	1.480	1.20	1.20	50	50
G	930	200	300	50	520	1.488	0.96	1.44	40	60
H	935	150	350	50	515	1.496	0.72	1.68	30	70
I	940	100	400	50	510	1.504	0.48	1.92	20	80
J	945	50	450	50	505	1.512	0.24	2.16	10	90
K	950	0	500	50	500	1.520	0	2.40	0	100

Stock solutions of TR (3.2 mM), EDA (9.6 mM), (*R*,*R*)-CHDA (9.6 mM) and TFA (19.2 mM) in certain volumes, as shown in the columns v(TR), v(EDA), v(CHDA) and v(TFA), were mixed in toluene to form the dynamic libraries containing designed ratios of TR, EDA, and CHDA. The mixtures were then introduced in a thermostated bath at 60 °C for 48 hours to give the samples A to K for further CD and HPLC characterizations.

Supplementary Table 3. Percentage ratios of HPLC fractions and calculated concentrations of free EDA and (*R*,*R*)-CHDA in equilibrium solutions

Sample[a]	A	B	C	D	E	F	G	H	I	J	K
$[\mathrm{TR}]_0$ / mM[a]	1.440	1.448	1.456	1.464	1.472	1.480	1.488	1.496	1.504	1.512	1.520
$[\mathrm{EDA}]_0$ / mM	2.40	2.16	1.92	1.68	1.44	1.20	0.96	0.72	0.48	0.24	0.00
$[\mathrm{CHDA}]_0$ / mM	0.00	0.24	0.48	0.72	0.96	1.20	1.44	1.68	1.92	2.16	2.40
Frac(TR)[b]	0.0146	0.0169	0.0144	0.0179	0.0154	0.0098	0.0118	0.0138	0.0085	0.0131	0.0088
Frac($\mathbf{2}^6$)	0.0000	0.0000	0.0000	0.0000	0.0109	0.0259	0.0683	0.1529	0.3047	0.5844	0.9912
Frac($\mathbf{1}^1\mathbf{2}^5$)	0.0000	0.0000	0.0033	0.0202	0.0575	0.1268	0.2260	0.3314	0.3983	0.3176	0.0000
Frac($\mathbf{1}^2\mathbf{2}^4$)	0.0000	0.0039	0.0291	0.0908	0.1765	0.2651	0.3163	0.3016	0.2170	0.0752	0.0000
Frac($\mathbf{1}^3\mathbf{2}^3$)	0.0000	0.0322	0.1196	0.2235	0.2892	0.2976	0.2409	0.1485	0.0628	0.0097	0.0000
Frac($\mathbf{1}^4\mathbf{2}^2$)	0.0000	0.1264	0.2621	0.3001	0.2653	0.1885	0.1036	0.0433	0.0088	0.0000	0.0000
Frac($\mathbf{1}^5\mathbf{2}^1$)	0.0000	0.2867	0.2959	0.2174	0.1313	0.0647	0.0256	0.0085	0.0000	0.0000	0.0000
Frac($\mathbf{1}^6$)	0.9855	0.5338	0.2757	0.1301	0.0539	0.0218	0.0076	0.0000	0.0000	0.0000	0.0000
$[\mathrm{TR}]_{\mathrm{octa}}$ /mM[c]	1.419	1.423	1.435	1.438	1.449	1.466	1.470	1.475	1.491	1.492	1.507
$[\mathrm{EDA}]_{\mathrm{octa}}$ /mM[d]	2.129	1.899	1.675	1.442	1.222	1.020	0.807	0.597	0.397	0.188	0.000
$[\mathrm{CHDA}]_{\mathrm{octa}}$ /mM[e]	0.000	0.236	0.477	0.715	0.953	1.178	1.399	1.616	1.840	2.050	2.260
$[\mathrm{TR}]_{\mathrm{eq}}$ /mM[f]	0.021	0.025	0.021	0.026	0.023	0.014	0.018	0.021	0.013	0.020	0.013
$[\mathrm{EDA}]_{\mathrm{eq}}$ /mM[g]	0.271	0.261	0.245	0.238	0.218	0.180	0.153	0.123	0.083	0.052	0.000
$[\mathrm{CHDA}]_{\mathrm{eq}}$ /mM[h]	0.000	0.004	0.003	0.005	0.007	0.022	0.041	0.064	0.080	0.110	0.140
Normalized $[\mathrm{EDA}]_{\mathrm{eq}}$[i]	1.000	0.961	0.902	0.876	0.805	0.662	0.564	0.454	0.306	0.192	0.000
Normalized $[\mathrm{CHDA}]_{\mathrm{eq}}$[j]	0.000	0.029	0.019	0.039	0.053	0.157	0.293	0.455	0.572	0.782	1.000

[a] Samples with designed concentrations of $[\mathrm{TR}]_0$, $[\mathrm{EDA}]_0$ and $[\mathrm{CHDA}]_0$ were prepared according to the procedure in Supplementary Table .

[b] Fractions of TR, $\mathbf{2}^6$, $\mathbf{1}^1\mathbf{2}^5$, $\mathbf{1}^2\mathbf{2}^4$, $\mathbf{1}^3\mathbf{2}^3$, $\mathbf{1}^4\mathbf{2}^2$, $\mathbf{1}^5\mathbf{2}^1$ and $\mathbf{1}^6$ were measured by HPLC.

[c] The concentration of TR fragment in octahedra was calculated from the equation: $[\mathrm{TR}]_{\mathrm{octa}} = [\mathrm{TR}]_0 \times (1\text{-Frac(TR)})$.

[d] The concentration of EDA fragment in octahedra was calculated from the equation: $[\mathrm{EDA}]_{\mathrm{octa}} = \frac{1}{4}[\mathrm{TR}]_0 \times (\mathrm{Frac}(\mathbf{1}^1\mathbf{2}^5) + 2\cdot\mathrm{Frac}(\mathbf{1}^2\mathbf{2}^4) + 3\cdot\mathrm{Frac}(\mathbf{1}^3\mathbf{2}^3) + 4\cdot\mathrm{Frac}(\mathbf{1}^4\mathbf{2}^2) + 5\cdot\mathrm{Frac}(\mathbf{1}^5\mathbf{2}^1) + 6\cdot\mathrm{Frac}(\mathbf{1}^6))$.

[e] The concentration of CHDA fragment in octahedra was calculated from the equation: $[\mathrm{CHDA}]_{\mathrm{octa}} = \frac{1}{4}[\mathrm{TR}]_0 \times (6\cdot\mathrm{Frac}(\mathbf{2}^6) + 5\cdot\mathrm{Frac}(\mathbf{1}^1\mathbf{2}^5) + 4\cdot\mathrm{Frac}(\mathbf{1}^2\mathbf{2}^4) + 3\cdot\mathrm{Frac}(\mathbf{1}^3\mathbf{2}^3) + 2\cdot\mathrm{Frac}(\mathbf{1}^4\mathbf{2}^2) + \mathrm{Frac}(\mathbf{1}^5\mathbf{2}^1))$.

[f] The concentration of free TR in equilibrium solution was calculated from the equation: $[\mathrm{TR}]_{\mathrm{eq}} = [\mathrm{TR}]_0 - [\mathrm{TR}]_{\mathrm{octa}}$.

[g] The concentration of free EDA in equilibrium solution was calculated from the equation: $[\mathrm{EDA}]_{\mathrm{eq}} = [\mathrm{EDA}]_0 - [\mathrm{EDA}]_{\mathrm{octa}}$.

[h] The concentration of free CHDA in equilibrium solution was calculated from the equation: $[\mathrm{CHDA}]_{\mathrm{eq}} = [\mathrm{CHDA}]_0 - [\mathrm{CHDA}]_{\mathrm{octa}}$.

[i] The concentrations of free EDA in Sample B to K were normalized by the concentration of free EDA in Sample A.

[j] The concentrations of free CHDA in Sample A to J were normalized by the concentration of free CHDA in Sample K.

Supplementary Table 4. Energies for optimized structures as calculated with VASP.

Structure	Energy (eV)
EDA monomer	-64.223
CHDA monomer	-123.381
(*CCCC*)-$\mathbf{1}^6$	-2848.050
(*AAAA*)-$\mathbf{1}^6$	-2848.022
$\mathbf{1}^5\mathbf{2}^1$	-2907.202
[a] Twisted $\mathbf{1}^5\mathbf{2}^1$	[b] -2906.083
[c] (*CCCC*)-$\mathbf{1}^5\mathbf{2}^1$-(*S*,*S*)-CHDA	[d] -2907.201
[e] (*CCCC*)-$\mathbf{1}^5\mathbf{2}^1$	[f] -2906.463
(*cis*)-$\mathbf{1}^4\mathbf{2}^2$	-2966.361
(*trans*)-$\mathbf{1}^4\mathbf{2}^2$	-2966.358
(*fac*)-$\mathbf{1}^3\mathbf{2}^3$	-3025.546
(*mer*)-$\mathbf{1}^3\mathbf{2}^3$	-3025.538
(*cis*)-$\mathbf{1}^2\mathbf{2}^4$	-3084.714
(*trans*)-$\mathbf{1}^2\mathbf{2}^4$	-3084.723
$\mathbf{1}^1\mathbf{2}^5$	-3143.894
$\mathbf{2}^6$	-3203.100

[a] The structure of twisted $\mathbf{1}^5\mathbf{2}^1$ contains three EDA-linked vertices in *c.a.* 60° gauche conformation, whereas all EDA-linked vertices in the normal $\mathbf{1}^5\mathbf{2}^1$ are in *c.a.* −60° gauche conformation, as shown in Supplementary Fig. 24.

[b] Difference between energy of $\mathbf{1}^5\mathbf{2}^1$ and twisted $\mathbf{1}^5\mathbf{2}^1$ corresponds to 1.119 eV, *i.e.*, 108.0 kJ/mol.

[c] Whereas all other structures comprise (*R*,*R*)-CHDA, and if not noted differently AAAA configuration, this structure, which is shown in Supplementary Fig. 19c, comprises (*S*,*S*)-CHDA and a *CCCC* configuration.

[d] Difference in energy between (*AAAA*)-$\mathbf{1}^5\mathbf{2}^1$-(*R*,*R*)-CHDA and (*CCCC*)-$\mathbf{1}^5\mathbf{2}^1$-(*S*,*S*)-CHDA corresponds to 0.0006 eV (0.06 kJ/mol), *i.e.*, within the accuracy of the method.

[e] Structure is shown in Supplementary Fig. 19a.

[f] Difference in energy between (*CCCC*)-$\mathbf{1}^5\mathbf{2}^1$ and (*AAAA*)-$\mathbf{1}^5\mathbf{2}^1$ corresponds to 0.739 eV, *i.e.*, 71.3 kJ/mol.

Supplementary Table 5. Full table of vertex angles and dihedrals in all DFT optimized structures.

**Structure**	**Vertex**	**N-C-C-N dihedral (°)**	**N-C-C angle (°)**	**C-C-N angle (°)**
EDA monomer	EDA	-54.52	110.14	110.14
CHDA monomer	CHDA	-58.07	109.60	109.64
(*CCCC*)-$\mathbf{1}^6$	EDA	58.66	109.35	109.40
	EDA	58.65	109.33	109.31
	EDA	58.72	109.36	109.37
	EDA	58.67	109.30	109.31
	EDA	58.75	109.38	109.31
	EDA	58.69	109.39	109.31
(*AAAA*)-$\mathbf{1}^6$	EDA	-58.60	109.32	109.37
	EDA	-58.63	109.38	109.32
	EDA	-58.61	109.36	109.38
	EDA	-58.66	109.38	109.35
	EDA	-58.69	109.38	109.38
	EDA	-58.70	109.37	109.35
$\mathbf{1}^5\mathbf{2}^1$	EDA	-58.61	109.21	109.33
	EDA	-58.79	109.38	109.34
	EDA	-58.90	109.31	109.29
	EDA	-58.82	109.33	109.36
	EDA	-58.55	109.29	109.28
	CHDA	-61.41	107.94	107.95
(*cis*)-$\mathbf{1}^4\mathbf{2}^2$	EDA	-58.35	109.32	109.25
	EDA	-58.73	109.51	109.47
	EDA	-58.32	109.30	109.22
	EDA	-58.73	109.51	109.43
	CHDA	-61.43	107.96	107.96
	CHDA	-61.43	107.97	107.94
(*trans*)-$\mathbf{1}^4\mathbf{2}^2$	EDA	-58.67	109.33	109.38
	EDA	-58.99	109.41	109.41
	EDA	-58.64	109.21	109.29
	EDA	-58.66	109.37	109.38
	CHDA	-61.50	107.98	108.01
	CHDA	-61.19	107.88	107.92
(*fac*)-$\mathbf{1}^3\mathbf{2}^3$	EDA	-58.64	109.23	109.24
	EDA	-58.66	109.30	109.19
	EDA	-58.77	109.23	109.29
	CHDA	-61.27	107.80	107.84
	CHDA	-61.26	107.86	107.87
	CHDA	-61.19	107.81	107.87
(*mer*)-$\mathbf{1}^3\mathbf{2}^3$	EDA	-58.44	109.40	109.35
	EDA	-58.95	109.36	109.41
	EDA	-58.49	109.34	109.33
	CHDA	-61.44	107.97	108.01
	CHDA	-61.40	107.87	107.84
	CHDA	-61.23	107.86	107.87
(*cis*)-$\mathbf{1}^2\mathbf{2}^4$	EDA	-58.44	109.31	109.29
	EDA	-58.49	109.28	109.28
	CHDA	-61.56	107.89	107.87
	CHDA	-61.56	107.94	107.90
	CHDA	-61.18	107.76	107.78
	CHDA	-61.19	107.76	107.75
(*trans*)-$\mathbf{1}^2\mathbf{2}^4$	EDA	-58.71	109.29	109.36
	EDA	-58.45	109.26	109.23
	CHDA	-61.37	107.90	107.81
	CHDA	-61.19	107.83	107.82
	CHDA	-61.43	107.97	107.94
	CHDA	-61.33	107.84	107.82
$\mathbf{1}^1\mathbf{2}^5$	EDA	-58.40	109.36	109.35
	CHDA	-61.14	107.86	107.84
	CHDA	-61.37	107.95	107.96
	CHDA	-61.34	107.93	107.91
	CHDA	-61.14	107.88	107.92
	CHDA	-61.19	107.91	107.86
$\mathbf{2}^6$	CHDA	-61.50	107.89	107.89
	CHDA	-61.49	107.89	107.92
	CHDA	-61.50	107.90	107.89
	CHDA	-61.44	107.88	107.86
	CHDA	-61.54	107.92	107.90
	CHDA	-61.51	107.91	107.85

Supplementary Table 6. Average number of electrons per atom for atoms "at the vertices" as calculated with DFT.

Vertex	C(EDA/CHDA)	N(EDA/CHDA)	C(TR)
EDA in $\mathbf{1}^6$	3.681	6.171	3.323
CHDA in $\mathbf{2}^6$	3.658	6.172	3.325
(*trans*)-EDA monomer	3.689	6.112	-
(*cis*)-EDA monomer	3.701	6.114	-
CHDA monomer	3.668	6.120	-

Supplementary Table 7. Bond critical values, ICOHP and ICOOP for specific bonds in free EDA, free CHDA, an EDA vertex and the CHDA edge in a $\mathbf{1}^5\mathbf{2}^1$ octahedron as calculated with DFT.

Structure	Bond	Electronic density (e^-/bohr^3)	Laplacian (-)	ICOHP (eV)	ICOOP (e^-)
EDA vertex	b1	1.629	-14.43	-7.152	0.3469
	b2	1.871	-18.04	-8.764	0.3509
	b3	1.871	-18.06	-8.760	0.3507
	b4	2.565	-13.09	-13.106	0.6379
	b5	2.565	-13.05	-13.101	0.6377
CHDA vertex	b1	1.614	-13.78	-6.871	0.3726
	b2	1.861	-17.78	-8.625	0.3774
	b3	1.861	-17.82	-8.629	0.3773
	b4	2.563	-13.17	-13.158	0.6390
	b5	2.564	-13.19	-13.150	0.6391
(*trans*)-EDA monomer	b1	1.661	-14.56	-6.862	0.352
	b2	1.788	-16.64	-8.117	0.353
	b3	1.788	-16.73	-8.210	0.353
(*cis*)-EDA monomer	b1	1.706	-15.92	-6.577	0.352
	b2	1.771	-16.29	-8.160	0.346
	b3	1.771	-16.42	-8.093	0.343
CHDA monomer	b1	1.662	-14.69	-6.451	0.359
	b2	1.771	-16.36	-8.199	0.369
	b3	1.788	-16.65	-8.045	0.368

Bond names as indicated in Supplementary Fig. 44. ICOHP (Integrated Crystal Orbital Hamiltonian Population) and ICOOP (Integrated Crystal Orbital Overlap Population) values have been calculated for the N-C bonds between EDA / CHDA and TR in an octahedron. The ICOHP indicates the strength (in eV) of a bond, while the ICOOP indicates the bond order (the difference between the number of bonding electrons and the number of antibonding electrons in a bond). Both ICOHP and ICOOP suggest that the N-C bond is stronger for the CHDA-TR case than for EDA-TR case. One could infer from this that the CHDA structure should be more stable.

Supplementary Methods

Synthesis of TR. The hexapropyl trialdehydetruxene (TR) was prepared using a similar procedure to that described previously for the homologous compound hexabutyl trialdehydetruxene[1,2]. Briefly, truxene (51.4 g, 150 mmol), dimethyl sulfoxide (500 mL), and potassium t-butoxide (101 g, 900 mmol) were reacted with n-Propyl bromide (112 g, 900 mmol) at 0 °C for 1 hour. The reaction was then returned to room temperature before adding another three batches of potassium t-butoxide (900 mmol × 3) and n-propyl bromide (900 mmol × 3). Water and ethyl acetate were used to quench the reaction, and the organic layer was extracted, dried, filtered and evaporated to give the product hexapropyl truxene as a white powder. The hexapropyl truxene (5.11 g, 10 mmol) was then reacted with bromine (2.5 mL) and anhydrous $FeCl_3$ (20 mg) at 0 °C for 24 hours before the reaction was quenched with saturated sodium thiosulfate aqueous solution. The organic layer was washed and evaporated, and the residue was recrystallized from methanol to give the hexapropyl tribromotruxene as a white powder. Then, the hexapropyl tribromotruxene (1.34 g, 1.8 mmol) was reacted with n-BuLi (6.6 mL, 15.8 mmol, 2.4 M in hexane) in anhydrous ethyl ether as the temperature slowly increased from −78 °C to room temperature for 1 hour. The sample was re-cooled to −78 °C again and DMF (1.3 g, 17.6 mmol) was slowly added. After 24 hours, the reaction was quenched with aqueous hydrochloric acid (2 M, 100 mL). The organic layer was washed with aqueous sodium chloride solution (20 mL × 3) and dried over sodium sulfate. The solvent was evaporated under vacuum and the solid was purified by column chromatography with hexane:DCM = 9:1 (v/v) to give TR as a white powder. The overall yield is about 65%.

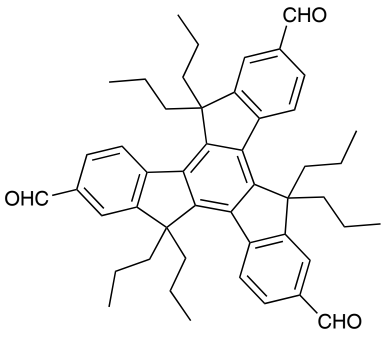

TR

TR. ^{1}H NMR (δ ppm, $CDCl_3$, 298 K, 500 MHz): 10.16 (s, 3H), 8.54 (d, *J*=8.15 Hz, 3H), 8.04 (d, *J*=1.50 Hz, 3H), 7.96 (dd, J_1=8.10 Hz, J_2=1.52 Hz, 3H), 2.87 – 2.92 (m, 6H), 2.21 – 2.24 (m, 6H), 0.53 (s, 30H); ^{13}C NMR (δ ppm, $CDCl_3$, 298 K, 126 MHz): 192.09, 154.36, 149.07, 145.51, 137.83, 134.94, 129.70, 124.94, 122.33, 54.24, 39.22, 17.38, 14.35. HRMS (m/z): $[M+H]^+$ calculated for $C_{48}H_{55}O_3$, 679.41457; found, 679.41400.

Synthesis of 1[6]. **TR** (100 mg, 0.1475 mmol), ethylenediamine (13.3 mg, 0.2212 mmol), and trifluoroacetic acid (1.261 mg, 0.0111 mmol) were reacted in toluene (100 mL) at room temperature

following the general procedure of synthesizing octahedra. Product: light solid , yield 95%.

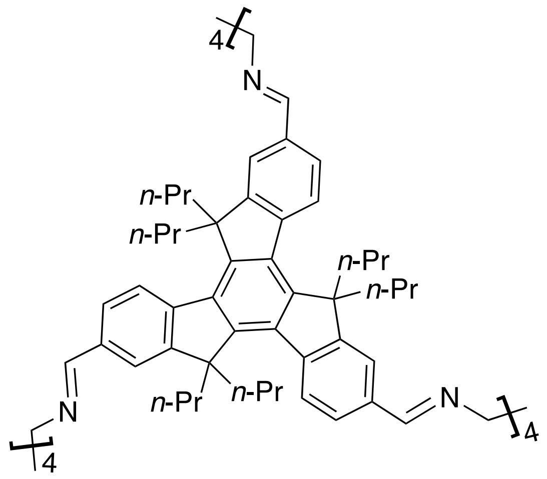

1[6]

1[6]. ^{1}H NMR (δ ppm, $CDCl_3$, 298 K, 600 MHz): 8.39 (s, 12H), 8.13 (d, *J*=8.21 Hz, 12H), 7.78 (d, *J*=1.54 Hz, 12H), 7.36 (dd, J_1=8.16 Hz, J_2=1.59 Hz, 12H), 4.37 (d, *J*=7.32 Hz, 12H), 3.91 (d, *J*=7.59 Hz, 12H), 2.84 – 2.70 (m, 12H), 2.56 – 2.40 (m, 12H), 2.04 – 1.87 (m, 12H), 1.61 – 1.56 (m, 12H), 0.40 – 0.24 (m, 48H), 0.23 – 0.11 (m, 12H), -0.05 – -0.22 (m, 12H), -0.23 – -0.37 (m, 36H), -0.38 – -0.48 (m, 12H). ^{13}C NMR (δ ppm, $CDCl_3$, 298 K, 150 MHz): 162.86, 154.37, 146.53, 142.65, 138.39, 135.21, 128.89, 125.00, 118.95, 61.74, 56.15, 41.36, 38.26, 17.70, 17.02, 14.57, 14.47. HRMS (m/z): $[M+2H]^{2+}$ calculated for $C_{228}H_{277}N_{12}$, 1592.10557; found, 1592.10571.

Synthesis of 2[6]**. TR** (100 mg, 0.1475 mmol), (*R,R*)-cyclohexane-1,2-diamine (25.2 mg, 0.2212 mmol), and trifluoroacetic acid (1.261 mg, 0.0111 mmol) were reacted in toluene (100 mL) at room temperature following the general procedure of synthesizing octahedra. The reaction was then heated up to 110 °C in a sealed tube for 48 hours to give the thermodynamic product (AAAA)-**2**[6]. Product: light solid , yield 97%.

2[6]. ^{1}H NMR (δ ppm, $CDCl_3$, 298 K, 600 MHz): 8.41 (s, 12H), 8.13 (d, *J*=8.19 Hz, 12H), 7.76 (d, *J*=8.23 Hz, 12H), 7.34 (dd, J_1=8.19 Hz, J_2=1.32 Hz, 12H), 3.56 – 3.40 (m, 12H), 2.89 – 2.72 (m, 12H), 2.58 – 2.34 (m, 12H), 2.03 – 1.80 (m, 48H), 1.59 – 1.58 (m, 24H), 0.41 – 0.24 (m, 48H), 0.22 – 0.07 (m, 12H), -0.06 – -0.20 (m, 12H), -0.26 – -0.36 (m, 36H), -0.37 – -0.51(m, 12H). ^{13}C NMR (δ ppm, $CDCl_3$, 298 K, 150 MHz): 160.83, 154.36, 146.46, 142.57, 138.43, 135.44, 128.99, 125.04, 118.82, 76.00, 56.19, 41.41, 38.27, 33.28, 25.20, 17.69, 17.02, 14.56, 14.53. HRMS (m/z): $[M+2H]^{2+}$ calculated for $C_{204}H_{242}N_{12}$, 1429.96472; found, 1429.96008.

Single-crystal X-ray diffraction. 1[6] was dissolved in mesitylene (0.5 mL) and layered with octane (2 mL) on top. Transparent block crystals were grown within three weeks. Single crystal X-ray diffraction data were collected on Rigaku SuperNova X-Ray single crystal diffractometer using

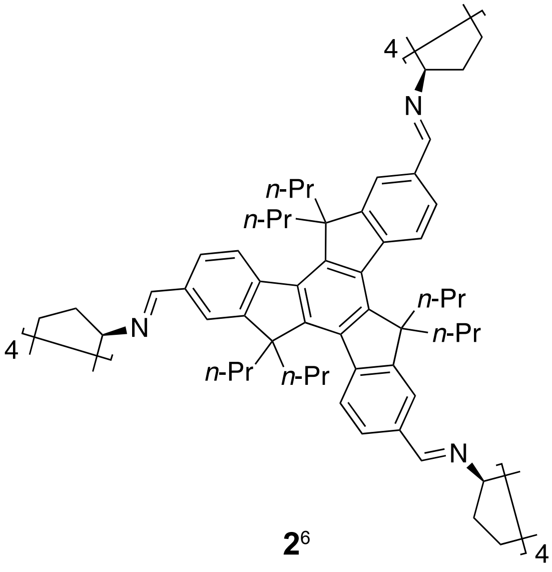

2[6]

Cu Kα (λ = 1.54184 Å) micro-focus X-ray sources. Suitable crystal was collected, covered with protective oil and mounted on X-ray diffractometer. The crystal was kept at 100 K with liquid nitrogen stream during the unit cell determination and full data collection. The raw data were collected and reduced by CrysAlisPro software, the structures were solved with the SHELXS[3] program using Direct Methods and refined with the SHELXL1 using CGLS minimization, and OLEX2[4] were used as GUI.

Refinement details: All non-hydrogen atoms were refined anisotropically. Hydrogen atoms were placed at calculated positions using the riding model and refined isotropically. The instructions AFIX 23 and AFIX 43 were used for the hydrogen atoms on the secondary -CH_2- and the aromatic C-H, respectively, with the parameter of U_{iso}=1.2 U_{eq}. The instruction AFIX 33 was used for the hydrogen atoms on the highly disordered terminal -CH_3 groups with the parameter of U_{iso}=1.5 U_{eq}. No Shelx restraint was applied to the skeleton of the octahedra, *i.e.*, truxene faces and diamine vertices. Nevertheless, the flexible propyl groups are expected to be highly disordered, as they are flexible and vibrate randomly in the large voids in the crystal. Therefore, necessary Shelx restraints (*i.e.*, DELU, SIMU, and EADP) were applied to the propyl groups to result in a reasonable model. Specifically, the anisotropic displacement parameters of disordered atoms in propyl groups were restrained to be equal within an effective standard deviation of 0.01 using the DELU command. U_{ij} values of disordered atoms of propyl groups were constrained to be similar using the SIMU command. Atomic displacement parameters (ADPs) of different parts of disordered atoms were restrained using the EADP command. There are large voids between the octahedra in crystal, filled with highly disordered solvent molecules. A satisfactory disorder model for the solvent molecules was not found, therefore the OLEX2 Solvent Mask routine (similar to PLATON/SQUEEZE) was used to mask out the disordered density. Note that racemic **1**[6] (*CCCC* & *AAAA*) are crystalized in non-symmetric space group P-43n, thus Flack parameter was reported.

Model for equilibrium distributions of mixed octahedra. The reaction scheme for the formation of mixed EDA/CHDA octahedra is illustrated in Fig. 5c. We assume 7 different octahedra $\mathbf{1}^n\mathbf{2}^m$ (with $0 \leq n, m \leq 6$ and $n+m=6$) and 6 reversible reactions in which EDA and CHDA are exchanged between the monomeric phase and the octahedra:

$$\mathbf{2}^6 + \text{EDA} \underset{k_1^-}{\overset{6k_1^+}{\rightleftharpoons}} \mathbf{1}^1\mathbf{2}^5 + \text{CHDA} \quad (1)$$

$$\mathbf{1}^1\mathbf{2}^5 + \text{EDA} \underset{2k_2^-}{\overset{5k_2^+}{\rightleftharpoons}} \mathbf{1}^2\mathbf{2}^4 + \text{CHDA} \quad (2)$$

$$\mathbf{1}^2\mathbf{2}^4 + \text{EDA} \underset{3k_3^-}{\overset{4k_3^+}{\rightleftharpoons}} \mathbf{1}^3\mathbf{2}^3 + \text{CHDA} \quad (3)$$

$$\mathbf{1}^3\mathbf{2}^3 + \text{EDA} \underset{4k_4^-}{\overset{3k_4^+}{\rightleftharpoons}} \mathbf{1}^4\mathbf{2}^2 + \text{CHDA} \quad (4)$$

$$\mathbf{1}^4\mathbf{2}^2 + \text{EDA} \underset{5k_5^-}{\overset{2k_5^+}{\rightleftharpoons}} \mathbf{1}^5\mathbf{2}^1 + \text{CHDA} \quad (5)$$

$$\mathbf{1}^5\mathbf{2}^1 + \text{EDA} \underset{6k_6^-}{\overset{k_6^+}{\rightleftharpoons}} \mathbf{1}^6 + \text{CHDA} \quad (6)$$

where we explicitly take into account that a $\mathbf{1}^n\mathbf{2}^m$ octahedron has n reactive sites for a CHDA and m for an EDA. Of course, other reaction pathways are possible, but as we are interested in equilibrium distributions, detailed balance states that also those reactions should be in equilibrium and addition of extra reaction pathways does not change the equilibrium constants (and corresponding free energy differences) of the reactions considered. Moreover, other complexes are ignored as they were not observed in the experiments.

Supplementary Eqs. 1–6 can be summarized by:

$$\mathbf{1}^{i-1}\mathbf{2}^{7-i} + \text{EDA} \underset{ik_i^-}{\overset{(7-i)k_i^+}{\rightleftharpoons}} \mathbf{1}^i\mathbf{2}^{6-i} + \text{CHDA} \quad (7)$$

for $1 \leq i \leq 6$. Because in equilibrium the fluxes in both directions should be equal, this yields

$$(7-i)k_i^+[\mathbf{1}^{i-1}\mathbf{2}^{7-i}]_{\text{eq}}[\text{EDA}]_{\text{eq}} = ik_i^-[\mathbf{1}^i\mathbf{2}^{6-i}]_{\text{eq}}[\text{CHDA}]_{\text{eq}} \quad (8)$$

where $[\text{x}]_{\text{eq}}$ denotes the equilibrium concentration of species x. This equation can be rewritten as

$$[\mathbf{1}^i\mathbf{2}^{6-i}]_{\text{eq}} = \frac{(7-i)}{i}K_i\frac{[\text{EDA}]_{\text{eq}}}{[\text{CHDA}]_{\text{eq}}}[\mathbf{1}^{i-1}\mathbf{2}^{7-i}]_{\text{eq}} \quad (9)$$

where $K_i = k_i^+/k_i^-$. This equation can be applied recursively to express the concentration of all octahedra types into the concentration of $[\mathbf{2}^6]_{\text{eq}}$, i.e.,

$$[\mathbf{1}^i\mathbf{2}^{6-i}]_{\text{eq}} = \frac{(7-i)\cdots 5\cdot 6}{i\cdots 2\cdot 1} K_i K_{i-1}\cdots K_1 \left(\frac{[\text{EDA}]_{\text{eq}}}{[\text{CHDA}]_{\text{eq}}}\right)^i [\mathbf{2}^6]_{\text{eq}} \tag{10}$$

for $1 \leq i \leq 6$. Using the multiplicative formula for binomial coefficients this can be rewritten as

$$[\mathbf{1}^i\mathbf{2}^{6-i}]_{\text{eq}} = \binom{6}{i}\left(\prod_{j=1}^{i} K_j\right)\left(\frac{[\text{EDA}]_{\text{eq}}}{[\text{CHDA}]_{\text{eq}}}\right)^i [\mathbf{2}^6]_{\text{eq}} \tag{11}$$

This implies that for a fixed set of equilibrium constants (K_i), equilibrium concentrations of all octahedra types are thus known as soon as $[\text{EDA}]_{\text{eq}}$, $[\text{CHDA}]_{\text{eq}}$ and $[\mathbf{2}^6]_{\text{eq}}$ are known.

Three mass balances can be devised, *i.e.*, one for the amount of TR, one for the amount of EDA, and one for the amount of CHDA.

First mass balance: for the overall concentration of TR ($[\text{TR}]_0$) we have

$$4([\mathbf{1}^6]_{\text{eq}} + [\mathbf{1}^5\mathbf{2}^1]_{\text{eq}} + [\mathbf{1}^4\mathbf{2}^2]_{\text{eq}} + [\mathbf{1}^3\mathbf{2}^3]_{\text{eq}} + [\mathbf{1}^2\mathbf{2}^4]_{\text{eq}} + [\mathbf{1}^1\mathbf{2}^5]_{\text{eq}} + [\mathbf{2}^6]_{\text{eq}}) + [\text{TR}]_{\text{eq}} = [\text{TR}]_0 \tag{12}$$

as each octahedron consists of 4 TR and no complexes other than octahedra are present.

Second mass balance: for the amount of EDA ($[\text{EDA}]_0$) we have

$$6[\mathbf{1}^6]_{\text{eq}} + 5[\mathbf{1}^5\mathbf{2}^1]_{\text{eq}} + 4[\mathbf{1}^4\mathbf{2}^2]_{\text{eq}} + 3[\mathbf{1}^3\mathbf{2}^3]_{\text{eq}} + 2[\mathbf{1}^2\mathbf{2}^4]_{\text{eq}} + 1[\mathbf{1}^1\mathbf{2}^5]_{\text{eq}} + [\text{EDA}]_{\text{eq}} = [\text{EDA}]_0 \tag{13}$$

Third mass balance: for the amount of CHDA ($[\text{CHDA}]_0$) we have

$$[\mathbf{1}^5\mathbf{2}^1]_{\text{eq}} + 2[\mathbf{1}^4\mathbf{2}^2]_{\text{eq}} + 3[\mathbf{1}^3\mathbf{2}^3]_{\text{eq}} + 4[\mathbf{1}^2\mathbf{2}^4]_{\text{eq}} + 5[\mathbf{1}^1\mathbf{2}^5]_{\text{eq}} + 6[\mathbf{2}^6]_{\text{eq}} + [\text{CHDA}]_{\text{eq}} = [\text{CHDA}]_0 \tag{14}$$

In Supplementary Eqs. 12–14, the concentrations $[\text{TR}]_0$, $[\text{EDA}]_0$ and $[\text{CHDA}]_0$ are known, and the equilibrium concentrations $[\text{TR}]_{\text{eq}}$ can be measured experimentally. Substituting the concentrations for the mixed polymers with the expressions in Supplementary Eq. 11 leaves three equations with three unknowns (i.e, $[\text{EDA}]_{\text{eq}}$, $[\text{CHDA}]_{\text{eq}}$ and $[\mathbf{2}^6]_{\text{eq}}$). We use the Matlab function lsqnonlin to solve these non-linear equations for a given set of equilibrium constants, thus providing concentrations for all octahedra types for that set of equilibrium constants.

The concentrations for all octahedra predicted by the model can then be compared to experimental data. A common measure for how well a model fits data is to take the sum of the squared differences between the experimental data points and the corresponding values predicted by the model. As a measure we thus define

$$err = \sum_{k\in M}\left([\mathbf{1}^i\mathbf{2}^{6-i}]_{\text{eq}}^{\text{exp},k} - [\mathbf{1}^i\mathbf{2}^{6-i}]_{\text{eq}}^{\text{model},k}\right)^2 \tag{15}$$

where M is the set of measurements at different CHDA/EDA ratios, $[\mathbf{1}^i\mathbf{2}^{6-i}]_{\text{eq}}^{\text{exp},k}$ is the experimentally measured concentration of octahedra $\mathbf{1}^i\mathbf{2}^{6-i}$ in measurement k, and $[\mathbf{1}^i\mathbf{2}^{6-i}]_{\text{eq}}^{\text{model},k}$ is the

concentration of octahedra $\mathbf{1}^i\mathbf{2}^{6-i}$ predicted by the model for that CHDA:EDA ratio. The error measure can then be minimized in a fit routine to determine for which set of six K_i values the model curves optimally fit the experimental data. With the overall TR, EDA and CHDA concentrations for the samples from A to K in Supplementary Table , the best fit obtained is shown in Supplementary fig. 5b.

For these fitted equilibrium constants, the curves representing the model results indeed seem to follow the experimental data closely, including the CD intensity (Supplementary Fig. 37) and the equilibrium concentrations of EDA and CHDA (Supplementary Fig. 39). The corresponding equilibrium constants are shown in Supplementary Fig. 38a. The corresponding predicted differences in free energies for the different octahedra are given in Supplementary Fig. 38b (as obtained using $K = \mathrm{e}^{-\Delta G/RT}$ and $T = 333$ K), relative to the free energy of $\mathbf{2}^6$.

Computational methods. Structures were constructed on the basis of the crystal structures and adjusted to follow the highest symmetries of T ($\mathbf{1}^6$ and $\mathbf{2}^6$), C_3 ((*fac*)-$\mathbf{1}^3\mathbf{2}^3$), D_2 ((*cis*)-$\mathbf{1}^4\mathbf{2}^2$ and (*cis*)-$\mathbf{1}^2\mathbf{2}^4$), C_2 ($\mathbf{1}^5\mathbf{2}^1$, $\mathbf{1}^1\mathbf{2}^5$ and (*mer*)-$\mathbf{1}^3\mathbf{2}^3$) and C_1 ((*trans*)-$\mathbf{1}^4\mathbf{2}^2$ and (*trans*)-$\mathbf{1}^2\mathbf{2}^4$). All structures were first optimized by the molecular mechanics method and further optimized by the DFT method. The molecular mechanics calculations were carried out in Materials Studios 7.0 as a licensed product of BIOVIA. Electrostatic potential energy between atoms arises from partial charges for each atom assigned by the COMPASS II force field[5,6]. Gasteiger charges and the smart algorithm were used within ultrafine calculation quality (2.0×10^{-5} kcal mol^{-1} energy, 0.001 kcal mol^{-1} Å^{-1} force, 1.0×10^{-5} Å displacement, with 5000 iterations for convergence tolerance). Structures optimized by COMPASS II force field were used as the initial structures, and further optimized by the DFT calculations to identify the stable configurations for all octahedron and diamine structures. First principle total energy calculations using the gradient corrected (PBE) density functional theory have been performed in vacuum, using the *ab initio* simulation code VASP[7–10]. The electronic wave functions have been expanded into plane waves up to an energy cut-off of 400 eV and a projector-augmented-wave (PAW) scheme[11] has been used in order to described the interactions between the valence electrons and the nuclei(ions). To calculate the octahedron structures in vacuum, large supercells (of the order of $39 \times 38 \times 39$ Å^3) have been employed. Depending on the structure, the number of atoms present per supercell ranges from 456 to 516. The minimum energy configurations have been considered to be reached when the forces on each atom of the structures were less than 0.025 eV/Å. Due to the large size of the supercells only one k-point, the Gamma point, was necessary to span the Brillouin zone. High accuracy settings (PREC=Accurate) have been enforced throughout. The possible van der Waals interactions have been considered through the Grimme D3-BJ method[12,13]. The energy barriers have been computed using the Climbing Image Nudged Elastic Band method[14,15]. Coordinates of all optimized structures are provided in a Supplementary Excel file. The CD spectra were calculated at ZINDO semi-empirical level[16,17] with Gaussian 09[18] using the energy-minimized structures optimized by the molecular mechanics and DFT calculations. To

generate the CD spectra, the electronic transitions were fitted to a Lorentzian distribution with the sigma value of 0.2 eV using GaussSum[19] as GUI.

Bonding and electronic structure analysis. The electronic structure and bonding analysis have been performed based on the partial density of states (PDOS), crystal orbital Hamiltonian population (COHP), and crystal orbital overlap population (COOP) functions. The PDOS is defined as

$$\mathrm{PDOS}_i(E) = \sum_n \langle \varphi_i \mid \Psi_n \rangle \, \delta(E - E_n) \, \langle \Psi_n \mid \varphi_i \rangle = \sum_n \mid c_i^n \mid^2 \, \delta(E - E_n) \tag{16}$$

where $\Psi_n = \sum_i c_i^n \varphi_i$ is built from the coefficients c_n^i and the atomic orbitals φ_i. It provides the probability to find an electron of energy E into an atomic orbital *i*, being also a measure of the degree of delocalization of the electron density, which relates to the interaction strength of the atomic orbitals on which the PDOS is constructed.

The crystal orbital Hamiltonian population is defined as

$$-\mathrm{COHP}_{ij}(E) = H_{ij} \sum_n c_i^n c_j^n \delta(E - E_n) \tag{17}$$

where H_{ij} is the Hamiltonian matrix element between atomic orbitals φ_i and φ_j. It measures the sign and magnitude of the bond order energy overlap between atomic orbitals located on different atoms[20–22]. This enables the determination of the bonding, anti-bonding or non-bonding character of the orbitals interaction, as well as the strength of the interaction. For positive values of -COHP_{ij}(E) the electronic interaction between the two atomic orbitals is bonding, negative values of -COHP_{ij}(E) describe an anti-bonding type of interaction, while a zero value is associated with the non-bonding interaction regime. The integrated value ICOHP_{ij} is equal to the contribution to the bond energy of the interaction of atomic orbitals *i* and *j*, apart from a correction due to differences in electrostatic interactions[23].

The crystal orbital overlap population density (COOP), defined by Hoffmann[24], is related to -COHP_{ij}(E), and is defined as:

$$\mathrm{COOP}_{ij}(E) = S_{ij} \sum_n c_i^n c_j^n \delta(E - E_n) \tag{18}$$

where $S_{ij} = \langle \varphi_i \mid \varphi_j \rangle$ is the overlap of atomic orbitals φ_i and φ_j. The values of COOP_{ij}(E) are also a measure for the bonding or antibonding character of an orbital fragment, but the bond order density is now weighted by the atomic orbital overlap S_{ij} instead of the bond energy overlap H_{ij}. Quantitative information on the bond characteristics can be obtained additionally from the Bader charges and the topological electron density properties such as the Laplacian and electron density at bond critical points[25–30]. The Bader charges are a measure of the electron occupation of an atom, and therefore offer information on the charge transfer. The electron density values at the bond critical point are a measure of the bond order of the chemical bond. It probes the covalent energy

contribution to the chemical bond. When the Laplacian value at the bond critical point is negative the bond can be considered covalent, when it is positive it has to be considered ionic.

Supplementary References

[1] Tsuji, H. *et al.* Tripyridyltruxenes: thermally stable cathode buffer materials for organic thin-film solar cells. *Asian J. Org. Chem.* **1**, 34–37 (2012).

[2] Wang, X. *et al.* Assembled molecular face-rotating polyhedra to transfer chirality from two to three dimensions. *Nature Commun.* **7**, 12469 (2016).

[3] Sheldrick, G. M. Crystal structure refinement with shelxl. *Acta Crystallogr. Sect. C* **71**, 3–8 (2015).

[4] Dolomanov, O. V., Bourhis, L. J., Gildea, R. J., Howard, J. A. & Puschmann, H. Olex2: a complete structure solution, refinement and analysis program. *J. Appl. Crystallogr.* **42**, 339–341 (2009).

[5] Sun, H. COMPASS: an *ab initio* force-field optimized for condensed-phase applications overview with details on alkane and benzene compounds. *J. Phys. Chem. B* **102**, 7338–7364 (1998).

[6] Sun, H., Ren, P. & Fried, J. The COMPASS force field: parameterization and validation for phosphazenes. *Comput. Theor. Polymer Sci.* **8**, 229–246 (1998).

[7] Kresse, G. & Hafner, J. *Ab initio* molecular dynamics for liquid metals. *Phys. Rev. B* **47**, 558 (1993).

[8] Kresse, G. & Furthmüller, J. Efficiency of *ab-initio* total energy calculations for metals and semiconductors using a plane-wave basis set. *Comput. Mater. Sci.* **6**, 15–50 (1996).

[9] Kresse, G. & Furthmüller, J. Efficient iterative schemes for *ab initio* total-energy calculations using a plane-wave basis set. *Phys. Rev. B* **54**, 11169 (1996).

[10] Kresse, G. & Joubert, D. From ultrasoft pseudopotentials to the projector augmented-wave method. *Phys. Rev. B* **59**, 1758 (1999).

[11] Blöchl, P. E. Projector augmented-wave method. *Phys. Rev. B* **50**, 17953 (1994).

[12] Grimme, S., Antony, J., Ehrlich, S. & Krieg, H. A consistent and accurate *ab initio* parametrization of density functional dispersion correction (DFT-D) for the 94 elements H–Pu. *J. Chem. Phys.* **132**, 154104 (2010).

[13] Grimme, S., Ehrlich, S. & Goerigk, L. Effect of the damping function in dispersion corrected density functional theory. *J. Comput. Chem.* **32**, 1456–1465 (2011).

[14] Henkelman, G. & Jónsson, H. Improved tangent estimate in the nudged elastic band method for finding minimum energy paths and saddle points. *J. Chem. Phys.* **113**, 9978–9985 (2000).

[15] Henkelman, G., Uberuaga, B. P. & Jónsson, H. A climbing image nudged elastic band method for finding saddle points and minimum energy paths. *J. Chem. Phys.* **113**, 9901–9904 (2000).

[16] Telfer, S. G., Tajima, N. & Kuroda, R. CD spectra of polynuclear complexes of diimine ligands: theoretical and experimental evidence for the importance of internuclear exciton coupling. *J. Am. Chem. Soc.* **126**, 1408–1418 (2004).

[17] Telfer, S. G., Tajima, N., Kuroda, R., Cantuel, M. & Piguet, C. CD spectra of d-f heterobimetallic helicates with segmental di-imine ligands. *Inorg. Chem.* **43**, 5302–5310 (2004).

[18] Frisch, M. *et al.* Gaussian 09, rev. B. 01. *Gaussian Inc., Wallingford CT* (2010).

[19] O'boyle, N. M., Tenderholt, A. L. & Langner, K. M. cclib: A library for package-independent computational chemistry algorithms. *J. Comput. Chem.* **29**, 839–845 (2008).

[20] Dronskowski, R. & Bloechl, P. E. Crystal orbital hamilton populations (COHP): energy-resolved visualization of chemical bonding in solids based on density-functional calculations. *J. Phys. Chem.* **97**, 8617–8624 (1993).

[21] Deringer, V. L., Tchougréeff, A. L. & Dronskowski, R. Crystal orbital hamilton population (COHP) analysis as projected from plane-wave basis sets. *J. Phys. Chem. A* **115**, 5461–5466 (2011).

[22] Maintz, S., Deringer, V. L., Tchougréeff, A. L. & Dronskowski, R. Analytic projection from plane-wave and paw wavefunctions and application to chemical-bonding analysis in solids. *J. Comput. Chem.* **34**, 2557–2567 (2013).

[23] Landrum, G. A. & Dronskowski, R. The orbital origins of magnetism: from atoms to molecules to ferromagnetic alloys. *Angew. Chem. Int. Ed.* **39**, 1560–1585 (2000).

[24] Hoffmann, R. Interaction of orbitals through space and through bonds. *Acc. Chem. Res.* **4**, 1–9 (1971).

[25] Tang, W., Sanville, E. & Henkelman, G. A grid-based Bader analysis algorithm without lattice bias. *J. Phys. Condens. Matter* **21**, 084204 (2009).

[26] Sanville, E., Kenny, S. D., Smith, R. & Henkelman, G. Improved grid-based algorithm for Bader charge allocation. *J. Comput. Chem.* **28**, 899–908 (2007).

[27] Henkelman, G., Arnaldsson, A. & Jónsson, H. A fast and robust algorithm for Bader decomposition of charge density. *Comput. Mater. Sci.* **36**, 354–360 (2006).

[28] Yu, M. & Trinkle, D. R. Accurate and efficient algorithm for Bader charge integration. *J. Chem. Phys.* **134**, 064111 (2011).

[29] Vega, D. & Almeida, D. Aim-uc: An application for qtaim analysis. *J. Comput. Methods Sci. Eng.* **14**, 131–136 (2014).

[30] Becke, A., Matta, C. F. & Boyd, R. J. *The quantum theory of atoms in molecules: from solid state to DNA and drug design* (John Wiley & Sons, 2007).
